# Verruculosins A–B, New Oligophenalenone Dimers from the Soft Coral-Derived Fungus *Talaromyces verruculosus*

**DOI:** 10.3390/md17090516

**Published:** 2019-09-02

**Authors:** Minghui Wang, Longhe Yang, Liubin Feng, Fan Hu, Fang Zhang, Jie Ren, Yan Qiu, Zhaokai Wang

**Affiliations:** 1 Technical Innovation Center for Utilization of Marine Biological Resources, Third Institute of Oceanography, Ministry of Natural Resources, Daxue Road 184, Xiamen 361000, China; 2 School of Nursing and Health, Qingdao Huanghai University, Linghai Road 1145, Qingdao 266427, China; 3 High-field NMR Center College of Chemistry and Chemical Engineering, Xiamen University, Siming South Road 422, Xiamen 361005, China; 4 Eye Institute of Xiamen University, Fujian Provincial Key Laboratory of Ophthalmology and Visual Science, School of Medicine, Xiamen University, Xiamen 361102, China

**Keywords:** *Talaromyces verruculosus*, oligophenalenone dimers, soft coral-derived fungus, secondary metabolites

## Abstract

In an effort to discover new bioactive anti-tumor lead compounds, a specific tyrosine phosphatase CDC25B and an Erb family receptor EGFR were selected as drug screening targets. This work led to the investigation of the soft coral-derived fungus *Talaromyces verruculosus* and identification of two new oligophenalenone dimers, verruculosins A–B (**1**–**2**), along with three known analogues, bacillisporin F (**3**), duclauxin (**4**), and xenoclauxin (**5**). Compound **1** was the first structure of the oligophenalenone dimer possessing a unique octacyclic skeleton. The detailed structures and absolute configurations of the new compounds were elucidated on the basis of spectroscopic data, X-ray crystallography, optical rotation, Electronic Circular Dichroism (ECD) analysis, and nuclear magnetic resonance (NMR) calculations. Among which, compounds **1**, **3**, and **5** exhibited modest inhibitory activity against CDC25B with IC_50_ values of 0.38 ± 0.03, 0.40 ± 0.02, and 0.26 ± 0.06 µM, respectively.

## 1. Introduction

Over the past 60 years, a large number of natural products have been discovered from marine fungi, possessing new skeletons, unique structures, and unexpected substituents. Many marine-derived natural products have been used as potential tools for biomedical research and development, such as cephalosporins, griseofulrins, and ergosterols. In marine ecosystems, there are complex relationships between marine fungi and marine organisms, including symbiosis, parasitism, and antibiotics. The epiphytic and endophytic fungi from marine invertebrates participate in marine organic matter decomposition and provide nutrition and protection for parasitic hosts, which increases the efficiency of discovering novel bioactive substances compared to soil microbes [1]. It was estimated that the proportion of bioactive constituents from marine microorganisms was 37.13%, which was much higher than the average proportion of 28.39% [2]. The evolution of fungi-derived structures has come up with satisfactory solutions for a variety of interesting biomedical problems and has attracted great interest among chemists [3]. Marine fungi have become a rich source of lead compounds with important biomedical significance.

Oligophenalenone dimers are a kind of natural metabolite of fungi, with a unique bis(oxaphenalenone) hetero-dimer structure, which have been shown to inhibit the proliferation of several tumor cell lines. Duclauxin is the first reported oligophenalenone dimer discovered from the *Penicillium* or *Talaromyces* species [4], which was highly active against murine leukemia L-1210 cells and exhibited strong inhibitory effects on mitochondrial respiration [5]. Other analogues, desacetylduclauxin and xenoclauxin, showed similar effects against leukemia cell proliferation via the inhibition of adenosine triphosphate (ATP) synthesis in mitochondria [6]. Bascillosporins A-H were isolated from the fungi *Talaromyces bacillosporus* [7] and *Talaromyces stipitatus* [8]. Among these compounds, Bascillosporin A exhibited high cytotoxicity against human breast cancer (MCF-7) cells and human non-small cell lung carcinoma (NCL-H460) cells [9]. Bascillosporins B, C, and H were moderately active against the above two cell lines and human cervical cancer (Hela) cells [8,9].

The number of reported natural oligophenalenones dimers is no more than 20 and most of them are characterized by a heptacyclic ring system. Duclauxamide A1 was obtained from *Penicillium manginii*, containing a special *N*-2-hydroxyethyl moiety [10]. A soil fungus *Talaromyces stipitatus* yielded two novel skelet on products, talaroketals A with a rare benzannulated 5,6-spiroketal ring system, and talaroketals B with a fused bicyclic furano-pyran moiety within the bis(oxaphenalenone) hetero-dimer structure [11]. Cleaved ring A has been shown in bacillisporin G, which originated from fungus *Talaromyces stipitatus* [8]. To broaden our research in novel anti-tumor lead compounds, we selected Cell Division Cycle 25B (CDC25B), a specific tyrosine phosphatase [12], and epidermal growth factor receptor (EGFR), an Erb family receptor [13], as screening targets. This led to the systematic isolation of soft coral-derived fungus *Talaromyces verruculosus* and the identification of a novel oligophenalenone dimer verruculosin A (**1**) with an octacyclic skeleton, which had never been reported in previous literature. A new analogue verruculosin B (**2**) along with the known natural products bacillisporin F (**3**) [8], duclauxin (**4**) [4], and xenoclauxin (**5**) [14] were also obtained and elucidated.

## 2. Results and Discussion

### 2.1. Structure Elucidation of the New Compounds

**Figure 1:**
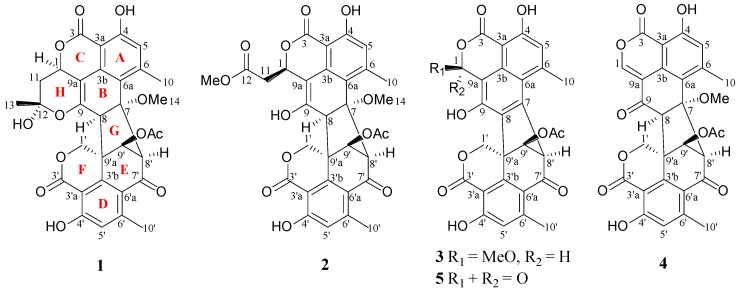
Structures of compounds **1**–**5**.

**Figure 2:**
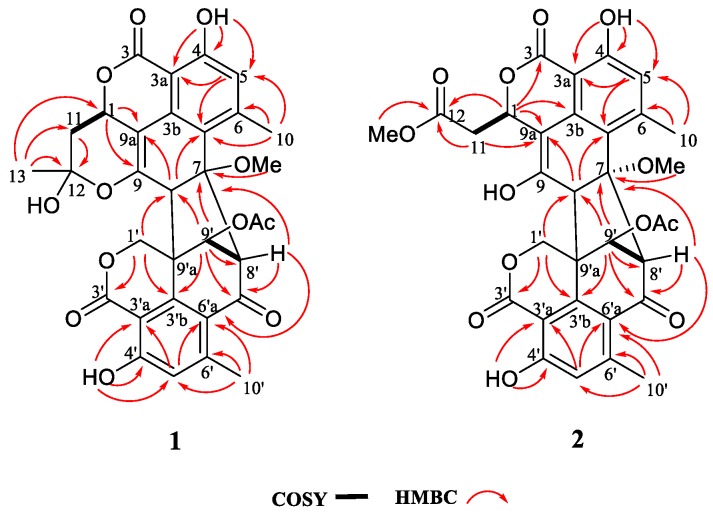
Key COSY and HMBC correlations of compounds **1**–**2**.

Verruculosin A (**1**) was obtained as a yellowish crystal. The high-resolution electrospray ionization-mass spectrometry (HR-ESI-MS) data recorded at *m/z* 605.1668 along with the analysis of the NMR data established the molecular formula of **1** as C_32_H_28_O_12_, indicating 19 degrees of unsaturation (DOU). The ^13^C and distortionless enhancement by polarization transfer (DEPT) NMR spectroscopic data (Table 1) exhibited the presence of 32 carbons, including five methyls, two methylenes, six methines, and 19 quaternary carbons. The heteronuclear multiple bond correlation (HMBC) correlations from H_2_-1′ to C-3′ (*δ*_C_ 168.0) and C-3′b (*δ*_C_ 143.5) proved the presence of a lactone ring F as shown (Figure 1 and Figure 2). Additionally, a six-membered ring E was elucidated by the ^1^H-^1^H homonuclear chemical shift correlation spectroscopy (COSY) relationship H-9′ (*δ*_H_ 5.17) with H-8′ (*δ*_H_ 4.16), and the HMBC correlations (Figure 2) from H-9′ to C-7′ (*δ*_C_ 190.9), C-8′ (*δ*_C_ 67.9), and C-3′b, from H-8′ to C-7′, C-6′a (*δ*_C_ 121.1), and C-9′a (*δ*_C_ 51.4). Observation of the correlations from H-5′ (*δ*_H_ 6.63) to C-3′a (*δ*_C_ 104.3) and C-6′a (*δ*_C_ 121.1) from H-10′ (*δ*_H_ 2.06) to C-5′, C-6′a, and C-6′ (*δ*_C_ 151.8) established a benzene ring D, which was fused to rings F and E via C-3′a, C-3′b, and C-6′a. Comparison of the abovementioned data with those of the duclauxin family [7] indicated that compound **1** was probably a dimer possessing two tricyclic moieties. Thus, the presence of another tricyclic moiety was further confirmed by the key HMBC correlations (Figure 2). Finally, the two tricyclic moieties were hinged by a five-membered ring G, which was strongly supported based on the key HMBC correlations (Figure 2) from H-8′, H-9′ to C-7, from H_2_-1′, H-9′, H-8′ to C-8 (*δ*_C_ 56.9), from H-8 to C-3′b (*δ*_C_ 143.5) and C-9′a. The established heptacyclic ring system (rings A–G), accounting for 17 of 19 DOU, and the remaining carbonyl group (*δ*_C_ 169.6) implied that one more ring should be present in the molecule. The deduction was supported by the ^1^H-^1^H COSY correlation between H_2_-11 and H-1 as well as the HMBC correlations from H_3_-13 (*δ*_H_ 1.74) to C-12 (*δ*_C_ 99.9), C-11 (*δ*_C_ 36.3), and C-1 (*δ*_C_ 69.1), from H_2_-11 (*δ*_H_ α 2.46, β 1.63) to C-12 and C-9a. Thus, the basic planar structure of **1** was determined.

**Figure 3:**
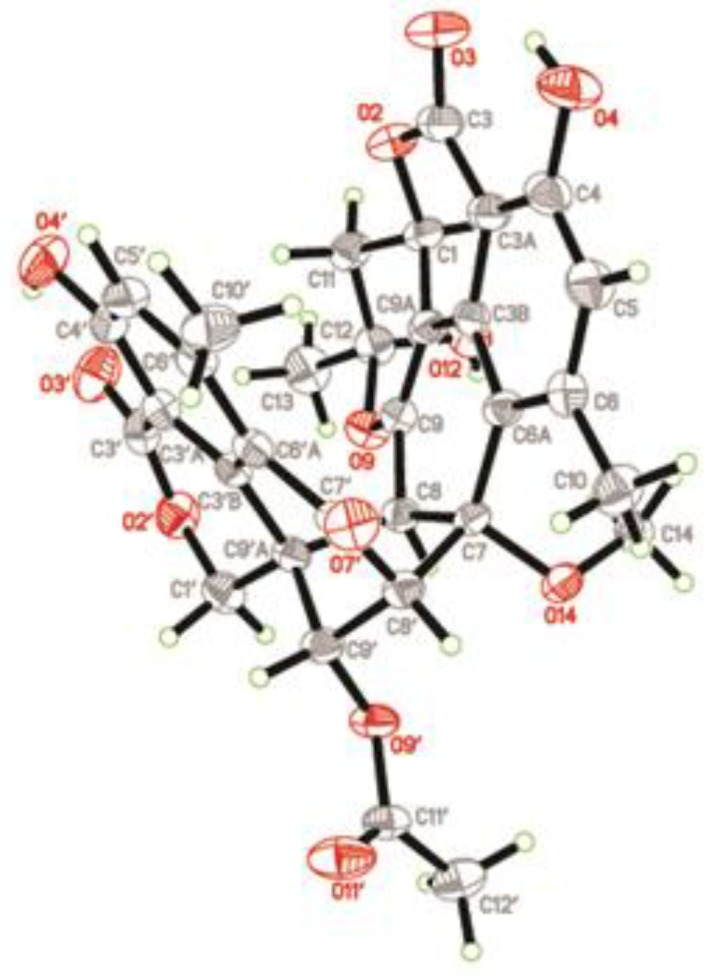
X-ray structure of compound **1**.

Since compound **1** was a novel oligophenalenone dimer possessing an octacyclic ring system, X-ray diffraction was necessary to confirm its structure and absolute configuration. To our delight, a high-quality crystal of **1** (Figure 3) was successfully obtained from CHCl_3_ and X-ray crystallographic analysis was carried out by applying the anomalous dispersion of Cu K*α* diffraction. Therefore, the absolute configurations of **1** were unambiguously assigned as 1*R*, 7*S*, 8*S*, 8′*S*, 9′S, 9′a*R*, and 12*S* by the refinement of the Flack parameter [0.13(11)]. The structure of **1** was thus elucidated as shown (Figure 1).

Verruculosin B (**2**) was obtained as a yellowish oil. Its molecular formula C_32_H_28_O_13_ was determined by HR-ESI-MS, with three carbon atoms, six proton atoms, and two oxygen atoms more than duclauxin (**4**). The ^1^H and ^13^C NMR data of **2** were similar to those of duclauxin (**4**) (Table 1), suggesting the same carbon skeleton for both compounds. The major difference was found by the presence of an additional moiety in **2**, which was assigned to a methoxycarbonylmethyl moiety based on the key HMBC correlations (Figure 2) from H_3_-13 (*δ*_H_ 3.65), H_2_-11 (*δ*_H_ α 2.70, β 2.93) to the ester carbonyl C-12 (*δ*_C_ 173.0). Moreover, the linkage of the methoxycarbonyl–methyl moiety to C-1 (*δ*_C_ 70.6) was confirmed by the HMBC correlations (Figure 2) from H_2_-11 to C-9a (*δ*_C_ 103.9) and from H-1 (*δ*_H_ 5.56) to C-12, along with the ^1^H-^1^H COSY relationship between H_2_-11 and H-1. Additionally, the resonance at *δ*_C_ 191.0 for a ketone group at the C-9 position in **4** was not present in the ^13^C NMR spectrum of **2** (Table 1). Rather, the signal indicative of a nonprotonated olefinic carbon at *δ*_C_ 150.9 (C-9) was observed in the ^13^C NMR spectrum of **2** (Table 1). These data indicated the replacement of the ketone group (C-9) in **4** by a hydroxylated olefinic carbon in **2**, which was consistent with the difference in the molecular formula.

**Figure 4:**
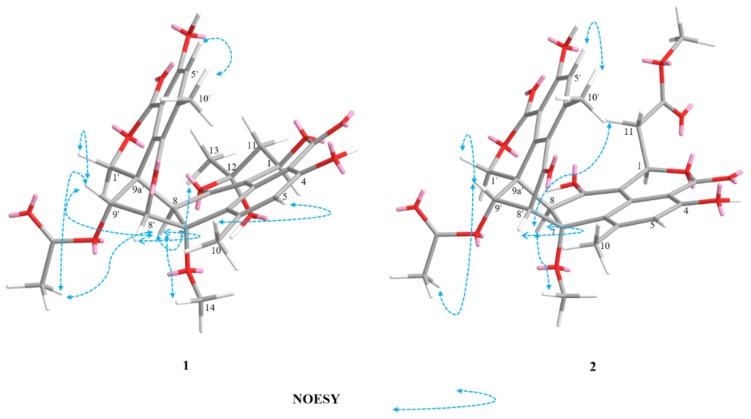
Key NOESY correlations of compounds **1**–**2**.

**Figure 5:**
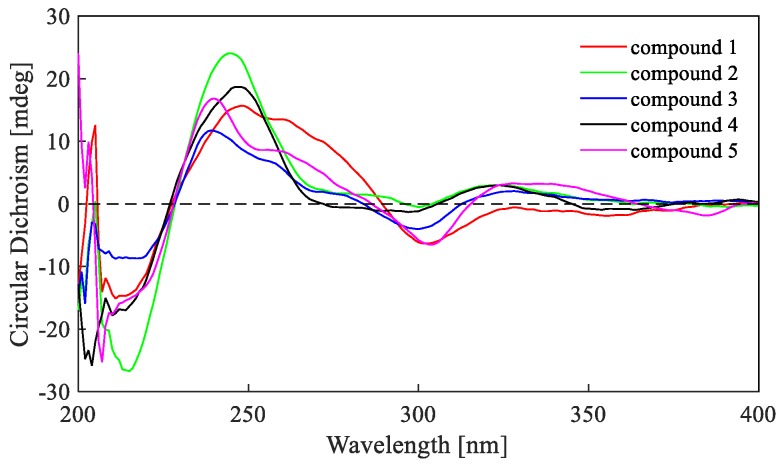
Electronic Circular Dichroism (ECD) spectra of compounds **1**–**5**.

The relative configurations of **2** were assigned the same as those of **1** on the basis of the nuclear overhauser effect spectroscopy (NOESY) correlations (Figure 4). Meanwhile, the ECD spectra (Figure 5) and optical rotation of **2** matched those of **1** and **4 [14,15]**. Therefore, we concluded that compound **2** possessed the same absolute configuration as verruculosin A (**1**) and duclauxin (**4**) [10]. In order to complement the above deduction for the configuration of C-1, we utilized NMR calculations [10,16,17].

The first step in making stereochemistry assignment was to do a conformational search, geometry optimization, and calculate NMR shielding constant, ^1^H, and ^13^C shifts for each candidate structure as described in computational methods (Supporting Information, S1). The next step was to match the experimental shifts with those of the calculated results, and values of mean absolute error (MAE), corrected mean absolute error (CMAE), maximum error (MaxErr), corrected maximum error (CMaxErr), the correlation coefficient, and DP4 probability were then calculated as described in the computational method (Supporting Information, S1). The results (Table 2) showed that it was not obvious regarding the correlation coefficient (*r*^2^ ˃ 0.99 in all cases), while the MAE, CMAE, MaxErr, and CMaxErr parameters of diastereomer 1*R* were better than those of diastereomer 1*S*. Moreover, the DP4 probability of diastereomer 1*R*, combining ^1^H and ^13^C values, was assigned as 100%, while those of the diastereomer 1*S* was assigned as 0%, suggesting *R*- configuration for C-1. Therefore, the absolute configuration of **2** was assigned as depicted (1*R*, 7*S*, 8*S*, 8′*S*, 9′S, 9′a*R*).

**Figure 6:**
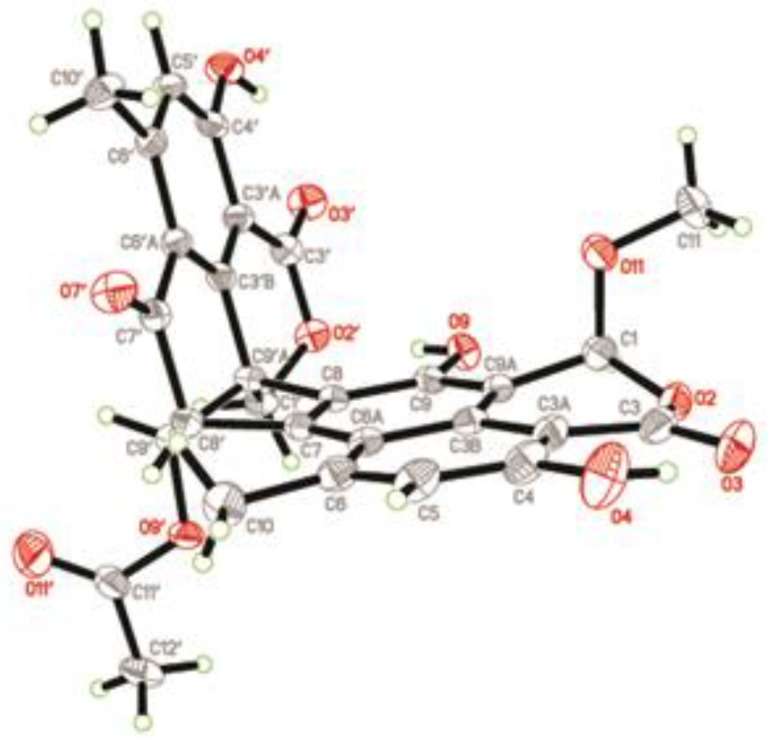
X-ray structure of compound **3**.

Bacillisporin F (**3**) was isolated as a yellowish crystal (from MeOH and CH_2_Cl_2_) with the molecular formula C_29_H_22_O_11_ as determined by HR-ESI-MS ([M − H]^–^ at *m/z* 545.1094). The structure of **3** was elucidated from NMR data, which was in accordance with that reported [8]. The absolute configurations of **3** were determined and confirmed by the single crystal X-ray diffraction analysis (Figure 6) for the first time. The final refinement of the Cu K*α* data resulted in a 0.05(14) Flack parameter, allowing an unambiguous assignment of the absolute configurations as 1*S*, 8′*R*, 9′a*S*, and 9′*S*.

In addition to compounds **1**–**3**, two known compounds, including duclauxin (**4**) [14,15] and xenoclauxin (**5**) [7] (Figure 1), were also isolated and elucidated by NMR and MS data comparison with the above literature.

### 2.2. Biological Activities of the Isolated Compounds

The crude extracts and isolated compounds were assayed for their inhibitory effects on EGFR [18] and CDC25B [12]. Despite their structural similarity, compounds **1**–**5** displayed remarkable differences in their activity spectra (Table 3). The EGFR tyrosine kinase assay showed that compounds **1**–**5** showed a weak inhibitory effect on EGFR. Besides, compounds **1**, **3**, and **5** displayed potent CDC25B inhibitory activities with IC_50_ values of 0.38 ± 0.03, 0.40 ± 0.02, and 0.26 ± 0.06 µM, respectively (Na_3_VO_4_, positive control, IC_50_ 0.52 ± 0.02 µM). The results indicated that oligophenalenone dimers might be used for screening as the new natural CDC25B inhibitor candidates.

## 3. Experimental Section

### 3.1. General

UV spectra were measured in methanol on a TU-1810 spectrophotometer (Beijing Purkinje General Instrument Company Limited, Beijing, China). X-ray crystallographic data were measured on a D/MAX-RC X-ray diffractometer (Rigaku Corporation, Krakow, Poland). Optical rotations were recorded in CHCl_3_ using a Rudolph Autopol III polarimeter (Rudolph Research Analytical, Hackettstown, NJ, USA) at 25 °C. Melting points were measured on a BUCHI M-560 melting point apparatus. NMR Spectra were recorded on a Bruker AVANCE III-600 MHz spectrometer (Bruker Corporation, Fällanden, Switzerland), and tetramethylsilane (TMS) was used as the internal standard. ECD spectra were measured on a JASCO J-810 circular dichroism spectrometer (JASCO Corporation, Tokyo, Japan) using MeOH as a solvent at 25 °C. HR-ESI-MS spectra were performed on a Xevo G_2_-QTOF spectrometer (Waters Corporation, Milford, MA, USA). Precoated silica gel plates (Qingdao Haiyang Chemical Group Co., Qingdao, China; GF_254_) were used for thin layer chromatography. Silica gel (Qingdao Haiyang Chemical Group Co., Qingdao, China; 100–200 mesh, 200–300 mesh), RP-18 reverse-phase silica gel (Silicycle, Quebec, Canada; 50 μm), and Sephadex LH-20 (GE Healthcare Bio-Sciences Corporation, Piscataway, NJ, USA) were used for column chromatography.

### 3.2. Fungal Material

The fungus was isolated from the soft coral *Goniopora* sp. collected from Sanya, Hainan island, South China Sea, China. The fungus was identified as *Talaromyces verruculosus* by analysis of the internal transcribed spacer (ITS) region sequence of its rDNA, as described previously [19]. The sequence data obtained from the fungus was submitted to GeneBank with accession number KU 057944. A voucher strain was deposited in the Technical Innovation Center for Utilization of Marine Biological Resources, Third Institute of Oceanography, Ministry of Natural Resources.

### 3.3. Fermentation

The fungus *Talaromyces verruculosus* was inoculated into 500 mL Erlenmeyer flasks containing 300 mL liquid medium (glucose 1%, maltose 2%, corn steep liquor 0.1%, yeast extract 0.3%, monosodium glutamate 1%, mannitol 2%, KH_2_PO_4_ 0.05%, MgSO_4_·7H_2_O 0.03%, seawater, pH 7.5). Static fermentation was then incubated at room temperature for 35 days.

### 3.4. Extraction and Isolation

The fermented whole broth (21 L) was filtrated to separate the supernatant from the mycelia. The supernatant was stirred three times with ethyl acetate (EtOAc) and was then concentrated under reduced pressure to obtain an EtOAc crude extract. The air-dried mycelia were immersed in acetone-H_2_O (4:1) with ultrasonic processor for 30 min before evaporating to afford an aqueous solution, which was extracted three times with EtOAc to yield a further EtOAc crude extract. Since both EtOAc extracts showed similar high performance liquid chromatography (HPLC) and thin layer chromatography (TLC) profiles, they were combined to afford an extract (27 g) for further purification. The extract was separated into seven fractions (Fr.1–Fr.7) on a silica gel column chromatography (CC) using a step gradient elution with MeOH–CH_2_Cl_2_ (0–100%). Fr.2 was further subjected to CC on Sephadex LH-20 (CH_2_Cl_2_–MeOH, 1:1) to obtain six subfractions (Fr.2.1–2.6). Furthermore, Fr.2.3 was purified by semi-preparative HPLC (35% acetonitrile) to yield **1** (30.1 mg, *t*_R_ 27.6 min), **3** (38.4 mg, *t*_R_ 29.8 min), and **4** (28.7 mg, *t*_R_ 28.0 min); Fr.2.4 was further purified by CC on Sephadex LH-20 (100% MeOH) and preparative TLC to obtain **5** (10.2 mg, *t*_R_ 32.3 min). Meanwhile, Fr.3 was subjected to CC on silica gel eluted with acetone–CH_2_Cl_2_ (2.5%–20%) and was then purified by Sephadex LH-20 CC (CH_2_Cl_2_–MeOH, 1:1) to get **2** (38.4 mg, *t*_R_ 31.3 min).

Verruculosin A (**1**): yellowish crystal (from CHCl_3_); m.p. 241–242 °C; ${\left[\alpha \right]}_{D}^{25}$ + 66 (*c* 0.37, CHCl_3_); UV (MeOH) λmax (log ε) 240 (4.53), 355 (3.62) nm; CD λmax 248 nm (Δε +38.3), 303 nm (Δε –15.5), 328 nm (Δε –1.3); ^1^H and ^13^C NMR data, see Table 1; HR-ESI-MS *m*/*z* 603.1519 [M – H]^–^ (calcd for C_32_H_27_O_12_, 603.1503), 627.1479 [M + Na]^+^ (calcd for C_32_H_28_O_12_Na, 627.1478). Crystal data (Cu K*α* radiation): C_32_H_28_O_12_, *M* = 604.54, monoclinic, *a* = 8.5126(2) Å, *b* = 17.3952(4) Å, *c* = 9.8690(3) Å, *α* = 90.00°, *β* = 113.780(4)°, *γ* = 90.00°, *V* = 1337.32(6) Å, *T* = 180.00(3) K, space group *P*1211, *Z* = 2, *µ* (Cu K*α*) = 0.979 mm^−1^, F (000) = 632. Crystal dimensions: 0.30 mm × 0.20 mm × 0.10 mm. *λ* (Cu K*α*) = 1.54184 Å. Further, 17,474 reflections were measured, with 3983 independent reflections (*R_int_* = 0.0376). The final *R*_1_ values were 0.0268 (*I* > 2*σ*(*I*)). The final *wR*(*F*^2^) values were 0.0666 (*I* > 2*σ*(*I*)). The final *R*_1_ values were 0.0281 (all data). The final *wR*(*F*^2^) values were 0.0681 (all data). The goodness of fit on *F*^2^ was 1.075. Flack parameter = 0.13(11).

Verruculosin B (**2**): yellowish oil; ${\left[\alpha \right]}_{D}^{25}$ + 110 (c 0.24, CHCl_3_); UV (MeOH) λmax (log ε) 236 (4.26), 316 (3.51), 367 (3.24) nm; CD λmax 246 nm (Δε +24.0), 300 nm (Δε –0.5), 323 nm (Δε +3.0); ^1^H and ^13^C NMR data, see Table 1; HR-ESI-MS *m*/*z* 619.1396 [M − H]^−^ (calcd for C_32_H_27_O_13_, 619.1452).

### 3.5. EGFR Activity Assay

The EGFR tyrosine kinase inhibitory activity was tested using enzyme-linked immunosorbent assay (ELISA). The compounds were dissolved in dimethyl sulfoxide (DMSO) buffer solution and were then distributed to a 96-well plate pre-coated with poly(Glu, Tyr)_4:1_ as a substrate. Each well was treated with 50 μL of 10 μM ATP solution diluted with reaction buffer and the reaction was started by the addition of EGFR tyrosine kinase. After 60 min of incubation at 37 °C, the plate was washed three times with phosphate buffered saline containing 0.1% Tween 20 (PBST). Next, antiphosphotyrosine (PY99) antibody 100 μL was added. After incubation for 30 min at 37 °C, the plate was washed three times and goat anti-mouse IgG horseradish peroxidase was added. The plate was reincubated at 37 °C for 30 min and washed as before. Lastly, 100 μL of color development solution was added and the plate was incubated at room temperature until color emerged. The reaction was terminated by adding 50 μL of 2 M H_2_SO_4_, and the absorbance was measured on the Molecular Devices SpectraMax 190 microplatereader at 490 nm [20]. The known EGFR inhibitor, afatibib, was utilized as a positive control, and 0.1% (*v*/*v*) DMSO was used as a negative control.

### 3.6. CDC25B Activity Assay

The enzymatic activity of the CDC25B was determined at 30 °C by monitoring the dephosphorylation of 3-*O*-methylfluorescein phosphate (OMFP), which was used as a substrate for the CDC25B and can be monitored at a 535 nm emission wavelength and 485 nm excitation wavelength in the EnVisionmultilabel plate reader [21]. The assay was measured in a 100 μL mixture system containing 50 mM Tris-HCl, pH 8.0, 50 mM NaCl, 10 μM OMFP, 100 nM CDC25B, 1 mM dithiothreitol (DTT), 1 mM ethylene diamine tetraacetic acid (EDTA), 1% glycerin, and 2 mg/L compound. The initial rate of the dephosphorylation was determined using the early linear region of the enzymatic reaction kinetic curve. DMSO and Na_3_VO_4_ were used as negative and positive controls, respectively.

## 4. Conclusions

Two new oligophenalenone dimers, verruculosins A–B (**1**–**2**), together with three known compounds (**3, 4**, **5**) were isolated and identified from the marine soft coral-derived fungus *Talaromyces verruculosus*. Compound **1** was the first compound possessing a novel octacyclic ring system in the oligophenalenone dimer family. The structures and absolute configurations of compounds **1** and **3** were confirmed by single-crystal X-ray diffraction analysis, while compound **2** was assigned on the basis of the optical rotation, ECD analysis, and NMR calculations. In the bioassay, compounds **1**, **3**, and **5** exhibited potent inhibition against CDC25B with IC_50_ values of 0.38 ± 0.03, 0.40 ± 0.02, and 0.26 ± 0.06 µM, respectively.

## Figures and Tables

**Table 1:** ^1^H (600 MHz) and ^13^C (150 MHz) NMR data for compounds **1**–**2** in CDCl_3_.

No.	1		2	
	*δ*_H_ (*J* in Hz)	*δ*_C_	*δ*_H_ (*J* in Hz)	*δ*_C_
1	5.06, ddd (10.2, 6.0, 2.4)	69.1, CH	5.56, dd (3.6, 10.2)	70.6, CH
3		169.5, C		167.9, C
3a		101.2, C		100.0, C
3b		134.6, C		134.1, C
4		161.9, C		161.7, C
5	6.55, s	117.9, CH	6.55, s	118.0, CH
6		149.0, C		149.5, C
6a		117.5, C		118.4, C
7		87.0, C		86.4, C
8	3.59, d (2.4)	56.9, CH	3.73, s	57.8, CH
9		147.5, C		150.9, C
9a		101.3, C		103.9, C
10	2.59, s	21.2, CH_3_	2.59, s	21.4, CH_3_
11	α 2.46, q (6.0) β 1.63, t (12.0)	36.3, CH_2_	α 2.70, m β 2.93, m	39.7, CH_2_
12		99.9, C		173.0, C
13	1.74, s	28.3, CH_3_	3.65, s	52.9, CH_3_
14	2.93, s	51.1, CH_3_	2.91, s	50.8, CH_3_
1′	α 4.94, d (12.0) β 4.85, d (12.0)	71.1, CH_2_	α 5.00, d (12.0) β 4.84, d (12.0)	71.2, CH_2_
3′		168.0, C		167.7, C
3′a		104.3, C		104.5, C
3′b		143.5, C		143.5, C
4′		164.4, C		164.4, C
5′	6.63, br.d	120.8, CH	6.62, s	120.8, CH
6′		151.8, C		151.4, C
6′a		121.1, C		121.1, C
7′		190.9, C		191.0, C
8′	4.16, s	67.9, CH	4.15, s	68.2, CH
9′	5.17, s	76.8, CH	5.16, s	76.7, CH
9′a		51.4, C		52.0, C
10′	2.06, s	22.2, CH_3_	2.07, s	22.1, CH_3_
4-OH	10.82, s		10.85, s	
4′-OH	11.71, s		11.63, s	
9-OH				
OAc		169.6, C		169.7, C
	2.20, s	21.0, CH_3_	2.21, s	21.0, CH_3_

**Table 2:** MAE, CMAE, MaxErr, CMaxErr, the correlation coefficient, and DP4 probability for **2**.

	*δ*_calc._ (1*R*)		*δ*_calc._ (1*S*)	
	^1^H	^13^C	^1^H	^13^C
MAE	0.2396	3.1123	0.2565	3.2633
CMAE	0.2225	1.9137	0.2559	2.1154
MaxErr	0.594	7.678	0.861	9.162
CMaxErr	1.100	5.337	1.132	4.394
*r* ^2^	0.9937	0.9989	0.992	0.9987
DP4	99.90%	98.10%	0.10%	1.90%
DP4 *	100.00%		0.00%	

The best agreements are highlighted in bold type; *r*^2^: correlation coefficient; MAE: mean average error; CMAE: corrected mean average error; MaxErr: maximum error; CmaxErr: corrected maximum error; * The data include carbon and proton data.

**Table 3:** The biological activities of compounds **1**–**5**.

Comp.	Anti-EGFRIC _50_ (µM) ± SD	Anti-CDC25BIC _50_ (µM) ± SD
**1**	0.92 ± 0.25	0.38 ± 0.03
**2**	1.22 ± 0.53	NT ^a^
**3**	4.41 ± 2.32	0.40 ± 0.02
**4**	0.95 ± 0.64	0.75 ± 0.18
**5**	0.24 ± 0.17	0.26 ± 0.06
Afatinib	0.0005 ± 0.00002	NT ^a^
Na_3_VO_4_	NT ^a^	0.52 ± 0.02

^a^ No test.

## Supplementary Materials

The materials are available online at https://www.mdpi.com/1660-3397/17/9/516/s1. Computational data, CIF files of **1** and **3**, as well as NMR and MS spectra for compounds **1**–**2**.
