# Supplementary material for: Verruculosins A–B, New Oligophenalenone Dimers from the Soft Coral-Derived Fungus Talaromyces verruculosus
Source: Mar Drugs. 2019 Sep 2;17(9):516. doi: 10.3390/md17090516 (PMC6780165; doi:10.3390/md17090516)

Supporting Information

Verruculosins A–B, New Oligophenalenone Dimers from the Soft Coral-Derived Fungus *Talaromyces verruculosus*

Minghui Wang [1,2,†], Longhe Yang [1,†], Liubin Feng [3], Fan Hu [1], Fang Zhang [1], Jie Ren [4], Yan Qiu [4,*] and Zhaokai Wang [1,*]

[1] Technical Innovation Center for Utilization of Marine Biological Resources, Third Institute of Oceanography, Ministry of Natural Resources, Daxue Road 184, Xiamen 361000, China

[2] School of Nursing and Health, Qingdao Huanghai University, Linghai Road 1145, Qingdao 266427, China

[3] High-field NMR Center College of Chemistry and Chemical Engineering, Xiamen University, Siming South Road 422, Xiamen 361005, China

[4] Eye Institute of Xiamen University, Fujian Provincial Key Laboratory of Ophthalmology and Visual Science, School of Medicine, Xiamen University, Xiamen 361102, China

* Correspondence: yanqiu@xmu.edu.cn (Y.Q.); wang@tio.org.cn (Z.W.); Tel.: +86-592-2188681 (Y.Q.); +86-592-2195562 (Z.W.)

† These authors contribute equally to this paper.

Content

S1: Computational methods.
S2: Theoretical chemical shift and geometries for verruculosin B (**2**).
References
S3: ^{1}H NMR (600 MHz, $CDCl_3$) spectrum of verruculosin A (**1**).
S4: ^{13}C NMR (150 MHz, $CDCl_3$) and DEPT spectra of verruculosin A (**1**).
S5: Expanded ^{13}C NMR (150 MHz, $CDCl_3$) and DEPT spectra of verruculosin A (**1**).
S6: Expanded ^{13}C NMR (150 MHz, $CDCl_3$) and DEPT spectra of verruculosin A (**1**).
S7: ^{1}H-^{1}H COSY NMR spectrum of verruculosin A (**1**).
S8: HSQC NMR spectrum of verruculosin A (**1**).
S9: HMBC NMR spectrum of verruculosin A (**1**).
S10: NOESY NMR spectrum of verruculosin A (**1**).
S11: ^{1}H NMR (600 MHz, $CDCl_3$) spectrum of verruculosin B (**2**).
S12: ^{13}C NMR (150 MHz, $CDCl_3$) and DEPT spectra of verruculosin B (**2**).
S13: ^{1}H-^{1}H COSY NMR spectrum of verruculosin B (**2**).
S14: HSQC NMR spectrum of verruculosin B (**2**).
S15: HMBC NMR spectrum of verruculosin B (**2**).
S16: NOESY NMR spectrum of verruculosin B (**2**).
S17: ^{1}H NMR (600 MHz, $CDCl_3$) spectrum of verruculosin C (**3**).
S18: ^{13}C NMR (150 MHz, $CDCl_3$) and DEPT spectra of verruculosin C (**3**).
S19: ^{1}H-^{1}H COSY NMR spectrum of verruculosin C (**3**).
S20: HSQC NMR spectrum of verruculosin C (**3**).
S21: HMBC NMR spectrum of verruculosin C (**3**).
S22: NOESY NMR spectrum of verruculosin C (**3**).

S1: Computational methods.

All the quantum mechanical calculations were carried out using Gaussian 09 [1] and displayed by Avogadro [2]. The RDKit with MMFFs force field was performed for the conformational analysis [3]. The conformational searches were done within 5 kcal mol^{-1} and with the large enough steps to find all low-energy conformers at least 5-10 times. The corresponding low-energy conformers were then used as the input structures for further geometry optimization with B3LYP/6-31G(d) method at the gas phase. For all optimized conformers, frequency calculations were performed to ensure the absence of imaginary frequency for energy-minimized conformers. The ^{1}H and ^{13}C NMR shielding tensors were calculated using GIAO (gauge-independent atomic orbital) technique with B3LYP method and 6-31G(d,p) basis set in the gas phase. The unscaled chemical shifts (δ_u) were computed as $\delta_u = \sigma^o - \sigma^x$, where σ^x was the Boltzmann averaged shielding tensor and σ^o was the shielding tensor for proton or carbon nuclei in TMS calculated at the same level of theory (31.7129 and 191.6329 ppm for ^{1}H and ^{13}C NMR chemical shifts, respectively). Boltzmann statistic based on relative free energies at 298.15 K was used in the computations.

The stereostructure assignment was based on the following methods [4].

Mean absolute error (MAE) was calculated as

$$\mathrm{MAE} = \frac{\sum_i^n \left|\delta_u^i - \delta_{exp}^i\right|}{n}$$

Corrected mean absolute error (CMAE). The data were empirically scaled according to $\delta_s = (\delta_s - \mathit{intercept})/\mathit{slope}$. The *slope* and *intercept* were calculated by the linear regression of δ_u against δ_{exp}. The purpose of this approach was to remove systematic errors in the shift calculation. CMAE was then calculated as

$$\mathrm{CMAE} = \frac{\sum_{\mathrm{i}}^{\mathrm{n}} \left|\delta_{\mathrm{s}}^{\mathrm{i}} - \delta_{\mathrm{exp}}^{\mathrm{i}}\right|}{n}$$

Max absolute error (MaxErr) showed the maximum absolute difference between the experimental and the calculated shifts (δ_u).

Corrected max absolute error (CMaxErr) showed the maximum absolute difference between the experimental and the scaled shifts (δ_s).

Correlation coefficient (r) suggested the correlation between calculated and experimental shifts.

DP4 probability. When only one set of experimental data was assigned to one of several possible diastereoisomers, the DP4 probability was applied to make accurate assignment among many diastereoisomers [5]. The detailed DP4 calculations see reference 5 in the supporting information.

S2: Theoretical chemical shifts and geometries for verruculosin B (2).

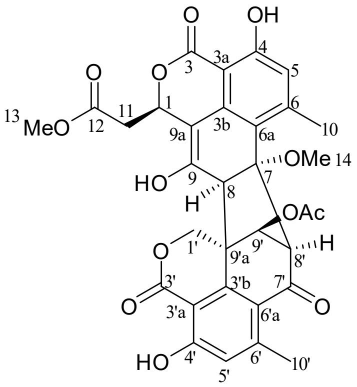

verruculosin B (1*R*)

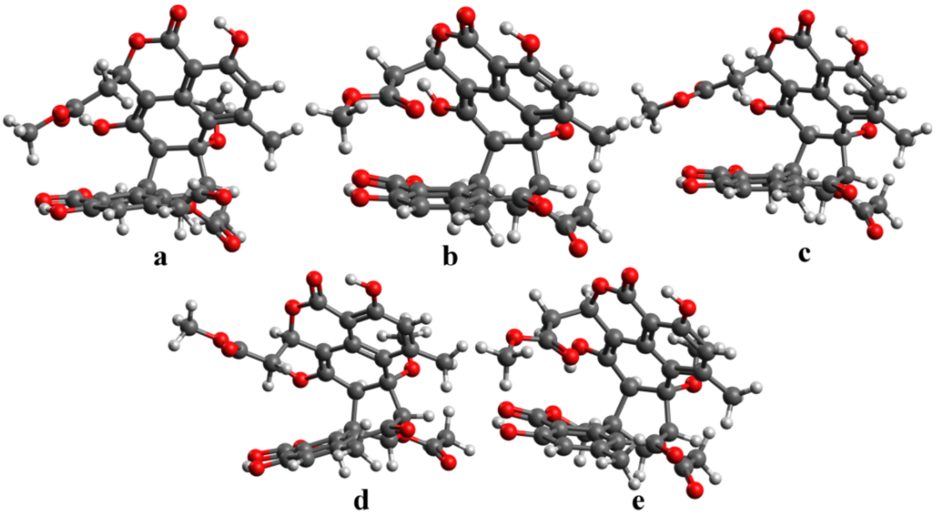

Figure S1. Optimized geometries of predominant conformers for verruculosin B (1*R*) at the B3LYP/6-31G(d) in the gas phase.

Table S1. Energies and Boltzmann distributions of the optimized verruculosin B (1*R*) at the B3LYP/6-31G(d) in the gas phase.

**Conformations**	**Relative Energy (kcal/mol)**	**Boltzmann mole fraction (%)**
verruculosin B (1*R*)**a**	0.02	49.11
verruculosin B (1*R*)**b**	7.83	0.00
verruculosin B (1*R*)**c**	0.00	50.81
verruculosin B (1*R*)**d**	5.42	0.01
verruculosin B (1*R*)**e**	3.89	0.07

Table S2. Optimized Z-matrixes of verruculosin B (1*R*) in the gas phase (Å) at B3LYP/6-31G(d) level.

verruculosin B (1*R*)**a**				verruculosin B (1*R*)**b**			
O	4.463761	8.998451	7.175555	O	4.361119	9.274635	6.826813
C	4.593182	10.335745	6.646875	C	4.326090	10.698667	6.990492
C	5.733827	11.100976	7.283856	C	5.633205	11.338143	7.429855

C	6.930985	10.341060	7.644371	C	6.859982	10.541914	7.512540
C	6.856331	8.925707	7.522378	C	6.774236	9.184067	7.117632
C	5.579409	8.246631	7.314821	C	5.511617	8.573368	6.712642
C	8.103223	10.957776	8.117800	C	8.063313	11.066093	8.028437
C	9.238861	10.146416	8.402727	C	9.181693	10.196288	8.177246
C	9.171982	8.768262	8.214688	C	9.092408	8.876301	7.747413
C	7.995055	8.134850	7.811747	C	7.915449	8.351420	7.208911
C	5.659693	12.437099	7.492461	C	5.626531	12.622225	7.845562
C	6.772763	13.234785	8.102915	C	6.848912	13.379617	8.230816
C	8.092753	12.449454	8.464235	C	8.135798	12.519370	8.508692
C	7.229813	14.487666	7.238941	C	7.307712	14.519475	7.233124
C	7.356422	14.124369	5.768208	C	7.438667	14.010924	5.804112
C	8.415867	13.309527	5.323422	C	8.547934	13.250563	5.397299
C	9.438042	12.876448	6.310360	C	9.594194	12.945424	6.401831
C	9.210669	13.300382	7.769761	C	9.282526	13.369669	7.843886
C	8.657496	14.723714	7.756936	C	8.723844	14.792344	7.762784
C	6.407829	15.772896	7.410867	C	6.467158	15.798799	7.247096
O	5.245770	15.911801	6.576717	O	5.242978	15.734228	6.492662
C	5.273851	15.470497	5.318282	C	5.229176	15.103657	5.312534
C	6.387136	14.609581	4.879327	C	6.405403	14.321486	4.905569
C	6.462977	14.238956	3.511437	C	6.477067	13.828146	3.576778
C	7.515897	13.424380	3.073642	C	7.605812	13.107756	3.168369
C	8.496791	12.959050	3.940291	C	8.638609	12.803782	4.042454
O	5.438949	7.024606	7.333843	O	5.403401	7.406425	6.342806
O	4.358627	15.802352	4.564650	O	4.216127	15.194771	4.616956
O	10.438149	12.239380	6.008085	O	10.666075	12.423440	6.127836
O	7.988674	6.797311	7.744318	O	7.914773	7.065427	6.836756
C	10.555988	10.694540	8.908582	C	10.500945	10.621905	8.786825
O	5.554930	14.638168	2.613394	O	5.496723	14.022068	2.687070
C	9.599469	12.099296	3.375355	C	9.800250	11.993626	3.527236
O	8.561561	15.328329	9.059084	O	8.601412	15.437064	9.044627
O	8.360545	12.623586	9.874015	O	8.439470	12.583285	9.918047
C	4.671477	10.198566	5.107082	C	3.693436	11.268332	5.702977
C	3.995696	11.326284	4.361433	C	4.454949	10.921067	4.436079
O	3.884111	11.055441	3.063751	O	3.639113	11.010286	3.371809
O	3.569531	12.355655	4.870168	O	5.628355	10.621810	4.380855
O	4.588385	13.211386	7.216102	O	4.507456	13.399422	7.976940
O	10.789879	15.724042	9.040449	O	10.818530	15.896447	9.025209
C	9.724713	15.769962	9.611535	C	9.750783	15.925256	9.591169

C	9.483821	16.294162	11.004536	C	9.489079	16.473757	10.970960
C	7.503443	11.933450	10.773463	C	7.542094	11.906113	10.786182
C	3.239457	12.057175	2.240575	C	4.241123	10.763266	2.085313
H	3.640203	10.802326	6.907291	H	3.581033	10.843250	7.789644
H	10.031355	8.143352	8.434428	H	9.942919	8.209431	7.844872
H	6.391374	13.654036	9.042160	H	6.624834	13.902503	9.167141
H	10.158614	13.221583	8.294068	H	10.201887	13.309703	8.420936
H	9.236674	15.387936	7.109294	H	9.313587	15.435088	7.103621
H	7.051516	16.636776	7.204103	H	7.062407	16.625978	6.839782
H	6.035647	15.865318	8.430461	H	6.160903	16.059415	8.259530
H	7.547536	13.166198	2.019734	H	7.636162	12.758898	2.141363
H	7.053376	6.524383	7.553911	H	6.996381	6.859120	6.520372
H	11.103544	11.173479	8.089535	H	11.098986	11.174466	8.054060
H	10.411332	11.431680	9.700829	H	10.357864	11.260545	9.660009
H	11.176631	9.882218	9.296923	H	11.073688	9.740071	9.087918
H	4.878654	15.175444	3.118667	H	4.762208	14.494984	3.164411
H	9.663579	11.137187	3.891446	H	9.933565	11.078944	4.112396
H	9.431066	11.924669	2.308468	H	9.636528	11.724805	2.479625
H	10.577687	12.570481	3.509277	H	10.741250	12.544103	3.613907
H	5.710354	10.121443	4.765029	H	2.673406	10.883588	5.608739
H	4.175095	9.267247	4.812314	H	3.616015	12.361939	5.751657
H	4.051594	12.843316	6.472792	H	3.698894	12.878660	7.854844
H	8.670796	17.026650	11.007125	H	9.203291	15.651275	11.635737
H	9.181803	15.463964	11.652232	H	10.391754	16.955293	11.347947
H	10.399523	16.745698	11.387270	H	8.659129	17.186872	10.953242
H	6.446673	12.202337	10.631854	H	6.532728	12.342852	10.760580
H	7.602348	10.845034	10.686794	H	7.471192	10.837029	10.552515
H	7.810809	12.243245	11.775334	H	7.948465	12.027116	11.793298
H	2.259918	12.308536	2.652300	H	4.836021	9.847992	2.112182
H	3.864188	12.950609	2.188475	H	3.406806	10.663620	1.390613
H	3.141227	11.594401	1.259114	H	4.870488	11.611095	1.805332
verruculosin B (1*R*)**c**				verruculosin B (1*R*)**d**			
O	4.889316	8.681745	6.896673	O	4.407061	9.414374	6.575063
C	4.799506	10.090147	6.605518	C	4.582576	10.828654	6.382339
C	5.882817	10.914913	7.271505	C	5.716839	11.420607	7.189011
C	7.172263	10.274965	7.539646	C	6.868660	10.572217	7.492693
C	7.272720	8.882989	7.263511	C	6.748076	9.192385	7.177233
C	6.097692	8.079486	6.937268	C	5.484617	8.609838	6.725466
C	8.264260	10.971869	8.094433	C	8.028191	11.074548	8.110630

C	9.472922	10.265099	8.355177	C	9.094233	10.174220	8.399388
C	9.573283	8.916753	8.024747	C	8.979411	8.831984	8.050265
C	8.495019	8.204636	7.497641	C	7.825069	8.315383	7.457080
C	5.669370	12.214999	7.589712	C	5.696820	12.722196	7.545414
C	6.736609	13.107133	8.136821	C	6.855020	13.436479	8.158550
C	8.107892	12.436033	8.514193	C	8.094283	12.540632	8.551843
C	7.095631	14.342979	7.206616	C	7.441450	14.622572	7.280475
C	7.332352	13.909809	5.766325	C	7.633680	14.192844	5.834094
C	8.500544	13.211169	5.408550	C	8.693070	13.342527	5.468970
C	9.539129	12.992221	6.447278	C	9.647001	12.911993	6.521656
C	9.166800	13.404622	7.877353	C	9.311083	13.337550	7.959467
C	8.458136	14.755045	7.787000	C	8.837135	14.788039	7.900230
C	6.129920	15.534771	7.261642	C	6.687671	15.956603	7.341736
O	5.002766	15.491548	6.367870	O	5.553640	16.083014	6.468060
C	5.138182	14.982370	5.144138	C	5.594052	15.547507	5.243744
C	6.352361	14.220550	4.808980	C	6.708022	14.653812	4.884989
C	6.535237	13.786073	3.467943	C	6.829799	14.219811	3.536984
C	7.688292	13.065758	3.127166	C	7.895315	13.381137	3.177698
C	8.678055	12.778917	4.057328	C	8.830500	12.939445	4.103193
O	6.121827	6.859996	6.773547	O	5.314030	7.407257	6.540840
O	4.227197	15.168752	4.333329	O	4.684490	15.821052	4.460706
O	10.652210	12.548007	6.202103	O	10.668274	12.279108	6.291660
O	8.656983	6.895215	7.270566	O	7.781184	7.002064	7.206346
C	10.696629	10.895947	8.985716	C	10.385835	10.589660	9.070469
O	5.643848	14.037068	2.505375	O	5.962606	14.574697	2.588115
C	9.893490	12.015437	3.595125	C	9.942449	12.040792	3.624206
O	8.220717	15.367874	9.067722	O	8.674814	15.399463	9.193112
O	8.318947	12.582124	9.934912	O	8.286953	12.630454	9.978590
C	4.740142	10.234180	5.063711	C	4.744410	11.102910	4.872554
C	3.747056	11.280015	4.604213	C	3.615995	10.522063	4.037169
O	3.623331	11.300137	3.280598	O	2.414310	10.931075	4.505687
O	3.104376	12.010872	5.347543	O	3.758917	9.810551	3.071328
O	4.504782	12.886775	7.469155	O	4.640484	13.565474	7.364550
O	10.378460	16.048528	9.143509	O	10.911839	15.721916	9.319194
C	9.290558	15.964568	9.664399	C	9.814572	15.806851	9.819577
C	8.917293	16.472591	11.033834	C	9.500311	16.349605	11.190594
C	7.489288	11.794751	10.778621	C	7.326227	11.966830	10.788910
C	2.684287	12.258282	2.736450	C	1.271613	10.420068	3.794281
H	3.822080	10.365747	7.009132	H	3.613817	11.229383	6.699814

H	10.491513	8.371890	8.218052	H	9.785600	8.139222	8.268399
H	6.347391	13.553577	9.059111	H	6.506768	13.908089	9.084725
H	10.078007	13.445977	8.467865	H	10.202663	13.209607	8.567360
H	8.994478	15.466931	7.153406	H	9.497269	15.417349	7.297104
H	6.681863	16.453535	7.028040	H	7.382001	16.768207	7.090631
H	5.695333	15.640481	8.254472	H	6.296384	16.140467	8.341716
H	7.791708	12.744327	2.095653	H	7.962476	13.075253	2.138562
H	7.769972	6.538762	7.000744	H	6.868091	6.802658	6.870758
H	11.254355	11.471418	8.238611	H	11.044710	11.077705	8.343714
H	10.431991	11.565915	9.805483	H	10.212996	11.283420	9.894974
H	11.361100	10.117310	9.370926	H	10.907644	9.709384	9.456168
H	4.904503	14.550997	2.933117	H	5.261051	15.127399	3.028903
H	10.068227	11.128157	4.210108	H	9.967764	11.101340	4.183787
H	9.771811	11.710760	2.551387	H	9.816374	11.820632	2.560205
H	10.799602	12.621446	3.686026	H	10.921155	12.504137	3.779591
H	5.722982	10.464103	4.637166	H	4.770926	12.188224	4.716445
H	4.424066	9.277280	4.632190	H	5.686430	10.687783	4.506765
H	3.872417	12.463903	6.839610	H	3.863952	13.093641	7.023109
H	8.019791	17.096722	10.983592	H	9.115646	15.539336	11.819371
H	8.690048	15.619921	11.682910	H	10.406551	16.762296	11.634650
H	9.749205	17.041266	11.450226	H	8.723912	17.118754	11.132776
H	6.422292	12.021945	10.641146	H	6.308722	12.351011	10.625576
H	7.643421	10.720637	10.621689	H	7.324071	10.883147	10.622500
H	7.772415	12.052549	11.802051	H	7.614287	12.171110	11.822905
H	1.663035	11.925506	2.937945	H	1.258422	9.328202	3.833784
H	2.845150	13.239897	3.182811	H	0.400955	10.835117	4.302366
H	2.881074	12.272068	1.665394	H	1.298528	10.741244	2.750060
verruculosin B (1*R*)**e**							
O	4.388256	9.178686	6.828411				
C	4.320694	10.609823	6.998427				
C	5.626046	11.272153	7.410300				
C	6.856208	10.489728	7.529210				
C	6.793580	9.125998	7.154885				
C	5.541039	8.493986	6.741103				
C	8.049512	11.039983	8.041645				
C	9.179720	10.189979	8.212482				
C	9.110825	8.861460	7.806557				
C	7.944893	8.311216	7.267988				
C	5.594621	12.573883	7.759353				

C	6.800137	13.348413	8.191094				
C	8.097845	12.500442	8.495690				
C	7.283693	14.508484	7.228177				
C	7.408165	14.028136	5.789880				
C	8.508766	13.266533	5.374075				
C	9.547708	12.930910	6.381266				
C	9.245308	13.347166	7.829182				
C	8.696392	14.773303	7.763880				
C	6.438142	15.775666	7.253634				
O	5.160629	15.599379	6.595824				
C	5.182457	15.093507	5.326151				
C	6.377045	14.368244	4.892989				
C	6.453937	13.908212	3.551600				
C	7.578744	13.186935	3.134725				
C	8.600898	12.848079	4.008860				
O	5.462627	7.319216	6.381597				
O	4.177406	15.239451	4.641120				
O	10.608776	12.392616	6.101146				
O	7.964533	7.020351	6.915094				
C	10.491481	10.642741	8.820181				
O	5.484223	14.130967	2.657991				
C	9.754030	12.033971	3.483127				
O	8.568142	15.406957	9.049711				
O	8.384732	12.595751	9.906697				
C	3.656109	11.154942	5.723248				
C	4.439089	10.889989	4.453788				
O	3.623558	10.951799	3.383120				
O	5.630606	10.668936	4.388238				
O	4.375888	13.201284	7.826652				
O	10.790394	15.839236	9.054740				
C	9.719352	15.873187	9.613619				
C	9.452826	16.403511	10.999146				
C	7.501563	11.899698	10.776379				
C	4.245334	10.758850	2.099048				
H	3.595699	10.753314	7.807050				
H	9.969600	8.207974	7.921479				
H	6.546418	13.848298	9.134582				
H	10.168330	13.277047	8.398467				
H	9.290642	15.420492	7.112788				

H	6.966669	16.596004	6.754233				
H	6.196718	16.084927	8.270572				
H	7.611692	12.860848	2.100482				
H	7.049345	6.800859	6.593094				
H	11.091831	11.178585	8.076970				
H	10.338837	11.302670	9.675860				
H	11.068401	9.773604	9.148989				
H	4.740174	14.584473	3.130352				
H	9.865343	11.102208	4.045563				
H	9.596945	11.795975	2.427288				
H	10.702795	12.566294	3.595087				
H	2.666006	10.700419	5.621465				
H	3.499109	12.233422	5.823419				
H	4.464752	14.156517	7.650309				
H	9.156402	15.572974	11.649081				
H	10.356668	16.872101	11.389332				
H	8.628636	17.123420	10.987293				
H	6.472406	12.282716	10.715200				
H	7.489483	10.822408	10.573877				
H	7.879211	12.071527	11.787251				
H	3.422737	10.629835	1.394959				
H	4.835715	11.639755	1.834272				
H	4.886025	9.874483	2.112940				

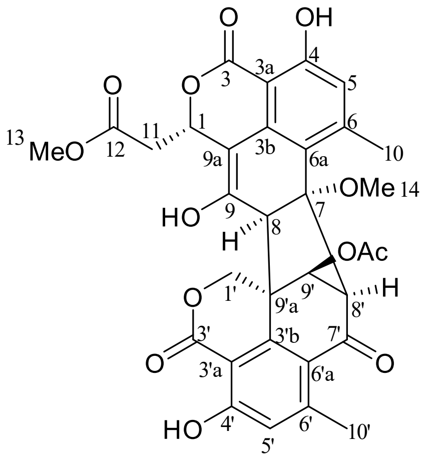

verruculosin B (1*S*)

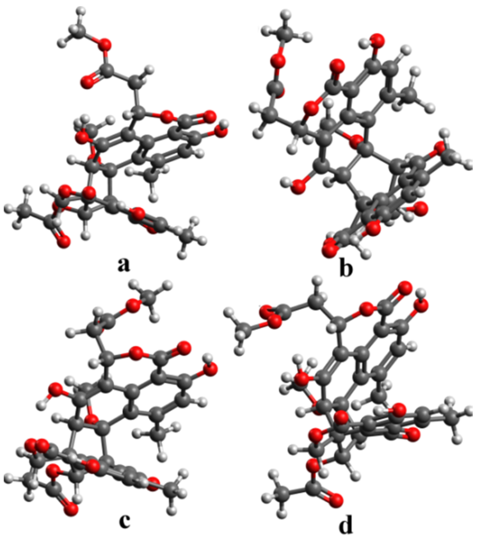

Figure S2. Optimized geometries of predominant conformers for verruculosin B (1*S*) at the B3LYP/6-31G(d) in the gas phase.

Table S3. Energies and Boltzmann distributions of the optimized verruculosin B (1*S*) at the B3LYP/6-31G(d) in the gas phase.

**Conformations**	**Relative Energy (kcal/mol)**	**Boltzmann mole fraction (%)**
verruculosin B (1*S*)**a**	0.00	99.29
verruculosin B (1*S*)**b**	6.97	0.00
verruculosin B (1*S*)**c**	3.09	0.53
verruculosin B (1*S*)**d**	3.74	0.18

Table S4. Optimized Z-matrixes of verruculosin B (1*S*) in the gas phase (Å) at B3LYP/6-31G(d) level.

verruculosin B (1*S*)**a**				verruculosin B (1*S*)**b**			
O	-2.693337	17.849679	12.248024	O	-2.979382	18.011770	12.533456
C	-2.879998	19.272469	12.424337	C	-3.374109	19.382240	12.371100
C	-2.205946	20.067219	11.328909	C	-2.613089	20.109764	11.285688
C	-0.896536	19.590479	10.884951	C	-1.217935	19.733854	11.058706
C	-0.499223	18.301152	11.328961	C	-0.770782	18.534988	11.670262
C	-1.451837	17.391544	11.962607	C	-1.690500	17.635680	12.365742
C	-0.039111	20.366378	10.081723	C	-0.339349	20.531332	10.303777
C	1.288739	19.907473	9.858463	C	1.037496	20.174735	10.260323
C	1.686749	18.666114	10.351755	C	1.471461	19.009475	10.889414
C	0.802031	17.826739	11.032340	C	0.590062	18.158230	11.559237
C	-2.739329	21.205017	10.822336	C	-3.155777	21.168567	10.650551
C	-2.075883	21.978405	9.728596	C	-2.420095	22.001210	9.656983
C	-0.553893	21.668921	9.463352	C	-0.860400	21.778972	9.582082
C	-2.776922	21.924885	8.313970	C	-2.941810	21.920846	8.166268
C	-3.026060	20.492612	7.861972	C	-3.090305	20.477832	7.708199
C	-1.979481	19.695632	7.363602	C	-1.965301	19.706084	7.368014
C	-0.626046	20.293642	7.265927	C	-0.627068	20.344381	7.436869
C	-0.460958	21.687263	7.890330	C	-0.583589	21.760081	8.032120
C	-1.665052	22.511461	7.430832	C	-1.749525	22.531998	7.414107
C	-4.063769	22.743517	8.177981	C	-4.222494	22.701854	7.858431
O	-5.248209	22.115181	8.694232	O	-5.449695	22.043691	8.217609
C	-5.445445	20.807296	8.483996	C	-5.576803	20.726750	8.012618
C	-4.332212	19.990611	7.973405	C	-4.385930	19.938473	7.658267
C	-4.586121	18.641087	7.608863	C	-4.551940	18.576479	7.289122
C	-3.539664	17.857417	7.100252	C	-3.426397	17.818202	6.935248
C	-2.250410	18.349894	6.962423	C	-2.143913	18.347126	6.958779
O	-1.213307	16.210204	12.206768	O	-1.376231	16.520859	12.774426

O	-6.555186	20.339909	8.734829	O	-6.693278	20.225536	8.144255
O	0.316828	19.755408	6.701571	O	0.397359	19.816634	7.026243
O	1.226717	16.605869	11.384881	O	1.075462	17.024947	12.083944
C	2.319346	20.692981	9.076194	C	2.090147	20.991528	9.542081
O	-5.790037	18.080862	7.726455	O	-5.744451	17.980419	7.261286
C	-1.186658	17.432783	6.413459	C	-0.993289	17.454475	6.568397
O	-1.550667	23.914569	7.736284	O	-1.723825	23.941939	7.703504
O	0.233491	22.805096	9.872500	O	-0.197277	22.968137	10.042742
C	-2.418077	19.604228	13.859401	C	-3.350656	20.102404	13.748198
C	-3.064613	20.840922	14.437054	C	-1.963401	20.444001	14.260627
O	-2.574627	21.136238	15.642879	O	-1.404720	19.394165	14.891875
O	-3.956479	21.489060	13.907656	O	-1.419255	21.518328	14.115504
O	-3.885564	21.791251	11.226191	O	-4.410307	21.658726	10.868708
O	-0.211844	24.201644	5.932635	O	-0.147290	24.242721	6.106878
C	-0.758500	24.654651	6.911150	C	-0.837034	24.693294	6.991613
C	-0.666737	26.077455	7.402764	C	-0.843130	26.120330	7.477637
C	0.316376	23.030832	11.271082	C	-0.287551	23.232111	11.440785
C	-3.162956	22.273132	16.310715	C	-0.059625	19.593191	15.367003
H	-3.963919	19.396831	12.368021	H	-4.432644	19.289718	12.095362
H	2.693878	18.303779	10.172158	H	2.517469	18.723231	10.847670
H	-2.119487	23.032663	10.021477	H	-2.560960	23.047017	9.952820
H	0.488981	22.099066	7.557387	H	0.384040	22.197499	7.799589
H	-1.862463	22.402059	6.361124	H	-1.811778	22.397361	6.330773
H	-4.240413	22.960806	7.116459	H	-4.265206	22.910706	6.781876
H	-3.987647	23.687934	8.715358	H	-4.243238	23.650518	8.393507
H	-3.770165	16.833187	6.825086	H	-3.589808	16.783915	6.649876
H	0.447376	16.132822	11.776459	H	0.302198	16.531371	12.463522
H	2.192912	20.506171	8.003498	H	2.095598	20.737494	8.475905
H	2.233037	21.767030	9.246265	H	1.910533	22.063224	9.637208
H	3.327061	20.369772	9.353668	H	3.081319	20.761896	9.944104
H	-6.385635	18.760319	8.145108	H	-6.408925	18.645629	7.587499
H	-1.603590	16.436872	6.237479	H	-0.242518	17.400606	7.362174
H	-0.773997	17.818201	5.476652	H	-1.356901	16.446013	6.350860
H	-0.339019	17.348693	7.099853	H	-0.469409	17.842791	5.690195
H	-2.686177	18.764431	14.513560	H	-3.892233	21.047521	13.645266
H	-1.328738	19.704510	13.915716	H	-3.870342	19.464381	14.469111
H	-4.115993	21.538051	12.149707	H	-4.934895	21.060761	11.424093

H	-1.665568	26.499727	7.550607	H	-1.862166	26.518835	7.496314
H	-0.155943	26.092200	8.371656	H	-0.458226	26.150675	8.502811
H	-0.108430	26.675667	6.682158	H	-0.212265	26.728324	6.828683
H	-0.665314	23.259816	11.711786	H	-1.324006	23.388207	11.768234
H	0.750033	22.172777	11.800233	H	0.141559	22.425416	12.043883
H	0.967970	23.898530	11.400456	H	0.278176	24.152052	11.606412
H	-3.028308	23.176368	15.711331	H	0.611007	19.795498	14.528087
H	-4.229413	22.104363	16.476420	H	-0.024369	20.430111	16.068661
H	-2.632265	22.354240	17.258619	H	0.213415	18.660968	15.860516
verruculosin B (1*S*)**c**				verruculosin B (1*S*)**d**			
O	-2.904636	17.936335	12.561008	O	-2.604326	17.717460	12.379707
C	-3.346571	19.297283	12.375311	C	-2.831482	19.135266	12.505990
C	-2.577626	20.037663	11.308238	C	-2.195884	19.925669	11.385486
C	-1.178003	19.693977	11.087322	C	-0.878505	19.479949	10.931656
C	-0.706587	18.508893	11.704802	C	-0.455533	18.194902	11.361456
C	-1.611670	17.594507	12.407202	C	-1.369200	17.276692	12.039236
C	-0.319589	20.503395	10.321457	C	-0.043693	20.284539	10.136494
C	1.062689	20.171227	10.268627	C	1.288581	19.848979	9.888177
C	1.520895	19.018535	10.903297	C	1.707305	18.603644	10.351222
C	0.659573	18.157034	11.588153	C	0.847912	17.744064	11.039730
C	-3.162651	21.072085	10.674267	C	-2.748597	21.061674	10.904908
C	-2.438950	21.943656	9.694010	C	-2.095916	21.899824	9.856340
C	-0.869122	21.740824	9.606886	C	-0.572017	21.606959	9.573143
C	-2.948962	21.903216	8.197779	C	-2.800215	21.927016	8.442182
C	-3.116927	20.471444	7.713796	C	-3.067894	20.521040	7.925739
C	-2.003355	19.700372	7.349830	C	-2.030085	19.733962	7.395822
C	-0.656177	20.324646	7.442338	C	-0.668702	20.319841	7.325222
C	-0.600139	21.733261	8.055733	C	-0.486861	21.687273	8.002547
C	-1.761072	22.522079	7.449581	C	-1.683849	22.541496	7.583726
C	-4.232883	22.677199	7.926177	C	-4.075310	22.768463	8.345046
O	-5.400676	22.014886	8.466366	O	-5.269062	22.133460	8.832643
C	-5.589340	20.709278	8.102918	C	-5.482320	20.838040	8.564545
C	-4.421963	19.944778	7.657781	C	-4.380846	20.031659	8.014809
C	-4.604062	18.599089	7.238444	C	-4.650835	18.702190	7.592367
C	-3.487436	17.843918	6.852792	C	-3.613474	17.928982	7.050481
C	-2.198447	18.356595	6.896343	C	-2.317558	18.410649	6.935410
O	-1.262929	16.491435	12.825705	O	-1.103200	16.101875	12.282837

O	-6.715898	20.245962	8.216865	O	-6.596471	20.373506	8.801351
O	0.363353	19.788975	7.032963	O	0.268802	19.790397	6.744352
O	1.168409	17.037280	12.118436	O	1.301140	16.530009	11.377191
C	2.096535	20.999790	9.535700	C	2.302003	20.666751	9.117053
O	-5.801098	18.012781	7.195929	O	-5.861283	18.151293	7.686378
C	-1.058447	17.464489	6.477001	C	-1.265250	17.506094	6.345738
O	-1.733702	23.927224	7.756951	O	-1.556601	23.927949	7.951118
O	-0.226151	22.941801	10.064677	O	0.206749	22.728458	10.029195
C	-3.375668	20.023377	13.746726	C	-2.348859	19.554092	13.915979
C	-2.011703	20.421355	14.273529	C	-2.725731	20.966548	14.311183
O	-1.429455	19.408463	14.947637	O	-4.060434	21.192621	14.115848
O	-1.489228	21.503940	14.103111	O	-1.978060	21.801704	14.758689
O	-4.431598	21.424738	11.041785	O	-3.915370	21.616062	11.326811
O	-0.144340	24.238683	6.175627	O	-0.206320	24.276434	6.167311
C	-0.834746	24.682699	7.062807	C	-0.749154	24.693460	7.163674
C	-0.830310	26.101243	7.571780	C	-0.634147	26.089074	7.722603
C	-0.292925	23.190123	11.468282	C	0.320476	22.874460	11.439655
C	-0.101277	19.666735	15.436774	C	-4.531181	22.499369	14.519454
H	-4.387677	19.196414	12.062789	H	-3.920743	19.214800	12.468636
H	2.571505	18.750287	10.856259	H	2.716972	18.258067	10.153874
H	-2.579647	22.981200	10.022340	H	-2.137906	22.935095	10.212142
H	0.370242	22.163825	7.822948	H	0.464722	22.102581	7.679872
H	-1.826453	22.401293	6.364548	H	-1.884702	22.480649	6.510722
H	-4.381880	22.801340	6.847248	H	-4.249059	23.036567	7.294913
H	-4.219898	23.662465	8.391610	H	-3.986070	23.686977	8.924034
H	-3.661893	16.821887	6.532408	H	-3.856360	16.920848	6.730263
H	0.403179	16.537922	12.510843	H	0.543328	16.044650	11.794559
H	2.098498	20.740235	8.470815	H	2.167245	20.509224	8.040777
H	1.902975	22.069624	9.627036	H	2.206084	21.734404	9.319662
H	3.094597	20.787930	9.930347	H	3.315953	20.347725	9.375572
H	-6.463068	18.653693	7.560161	H	-6.449197	18.817473	8.134861
H	-0.315361	17.367482	7.273928	H	-0.418611	17.380908	7.027000
H	-1.434209	16.471468	6.214715	H	-1.694149	16.524507	6.124289
H	-0.521640	17.882547	5.620496	H	-0.847664	17.929384	5.427585
H	-3.960734	20.937102	13.618491	H	-2.794912	18.862348	14.641322
H	-3.877421	19.365530	14.462320	H	-1.261794	19.473894	13.985876
H	-4.891093	21.881232	10.311628	H	-4.236100	21.195639	12.146379

H	-0.443566	26.111113	8.596666	H	-0.115108	26.048476	8.686399
H	-0.195415	26.715012	6.932307	H	-0.072557	26.713798	7.027520
H	-1.846072	26.507545	7.598542	H	-1.625472	26.517543	7.899762
H	-1.325941	23.293432	11.824661	H	-0.657159	22.996341	11.927397
H	0.187806	22.398700	12.051774	H	0.836181	22.023365	11.900301
H	0.234855	24.133541	11.626879	H	0.908492	23.780857	11.601865
H	0.195381	18.759874	15.963281	H	-5.608970	22.480686	14.359326
H	0.577558	19.866680	14.603606	H	-4.297149	22.671455	15.571566
H	-0.103983	20.525372	16.112924	H	-4.061974	23.273969	13.908751

Table S5. Comparison of the Calculated vs. Experimental Carbon Resonances for verruculosin B.

**No.**	$\delta_{\text{expt.}}$	$\delta_{\text{calcd.}}$ (***R***)	$\delta_{\text{calcd.}}$ (***S***)
1	70.63	72.9639	75.0399
3	167.92	162.2459	162.4392
3a	100.00	99.2039	99.3296
3b	134.09	132.0977	131.2185
4	161.71	158.7556	158.2182
5	118.01	114.4351	113.9516
6	149.52	146.8688	146.6661
6a	118.44	111.463	112.1541
7	86.35	89.3373	89.4974
8	57.80	60.7735	61.0655
9	150.86	145.4432	143.3249
9a	103.93	106.1772	105.6258
10	21.36	23.7548	22.8485
11	39.65	41.4338	41.129
12	172.99	166.9078	163.8276
13	52.93	51.6018	50.7588
14	50.78	48.816	50.9347
1'	71.18	72.7502	72.6829
3'	167.67	163.0075	162.9033
3'a	104.49	102.8801	101.9659
3'b	143.50	143.8188	144.132
4'	164.35	159.803	160.6292
5'	120.80	116.7965	116.9079
6'	151.44	150.5195	151.2701
6'a	121.14	118.1717	118.0211
7'	190.96	186.808	186.3034

8'	68.19	68.9508	69.0877
9'	76.71	81.1778	79.6703
9'a	51.95	54.9099	54.937
10'	22.14	26.39	26.1753
OAc	169.69	162.012	161.9703
OAc	20.99	20.3961	20.3697

Table S6. Comparison of the Calculated vs. Experimental Proton Resonances for verruculosin B.

No.	$\delta_{expt.}$	$\delta_{calcd.}$ (*R*)	$\delta_{calcd.}$ (*S*)
1	5.564	5.3599	5.482
5	6.552	6.4744	6.4509
8	3.726	3.6375	3.662
10	2.586	2.7062	2.6628
11α	2.700	2.3949	2.4052
11β	2.9145	2.3949	2.4052
13	3.655	3.725	3.1876
14	2.909	2.8839	3.0938
4-OH	10.849	11.9491	11.9812
1'α	5.004	4.6314	4.5805
1'β	4.8445	4.6314	4.5805
5'	6.619	6.6017	6.6303
8'	4.146	4.2534	4.1855
9'	5.164	4.6307	4.6282
10'	2.071	2.3695	2.2612
OAc	2.207	1.9529	1.9737
4'-OH	11.633	12.2598	11.6431

References

1. Frisch, M.J.; Trucks, G.W.; Schlegel, H.B.; Scuseria, G.E.; Robb, M.A.; Cheeseman, J.R.; Scalmani, G.; Barone, V.; Mennucci, B.; Petersson, G.A.; *et al*. *Gaussian 09*, Revision E.01; Gaussian, Inc.: Wallingford, CT, USA, **2009**.
2. Hanwell, M.D.; Curtis, D.E.; Lonie, D.C.; Vandermeersch, T.; Zurek, E.; Hutchison, G.R. Avogadro: an advanced semantic chemical editor, visualization, and analysis platform . *J. Cheminfor.* **2012**, *4*, 1–17.
3. RDKit: Cheminformatics and Machine Learning Software. **2013** [http://www.rdkit.org].
4. Smith, S.G; Goodman, J.M. Assigning the Stereochemistry of Pairs of Diastereoisomers Using GIAO NMR Shift Calculation. *J. Org. Chem*. **2009**, *74*, 4597–4607.
5. Smith, S.G; Goodman, J.M. Assigning Stereochemistry to Single Diastereoisomers by GIAO NMR Calculation: The DP4 Probability. *J. Am. Chem. Soc*. **2010**, *132*, 12946–12959.

S3: ^{1}H NMR (600 MHz, $CDCl_3$) spectrum of verruculosin A (1).

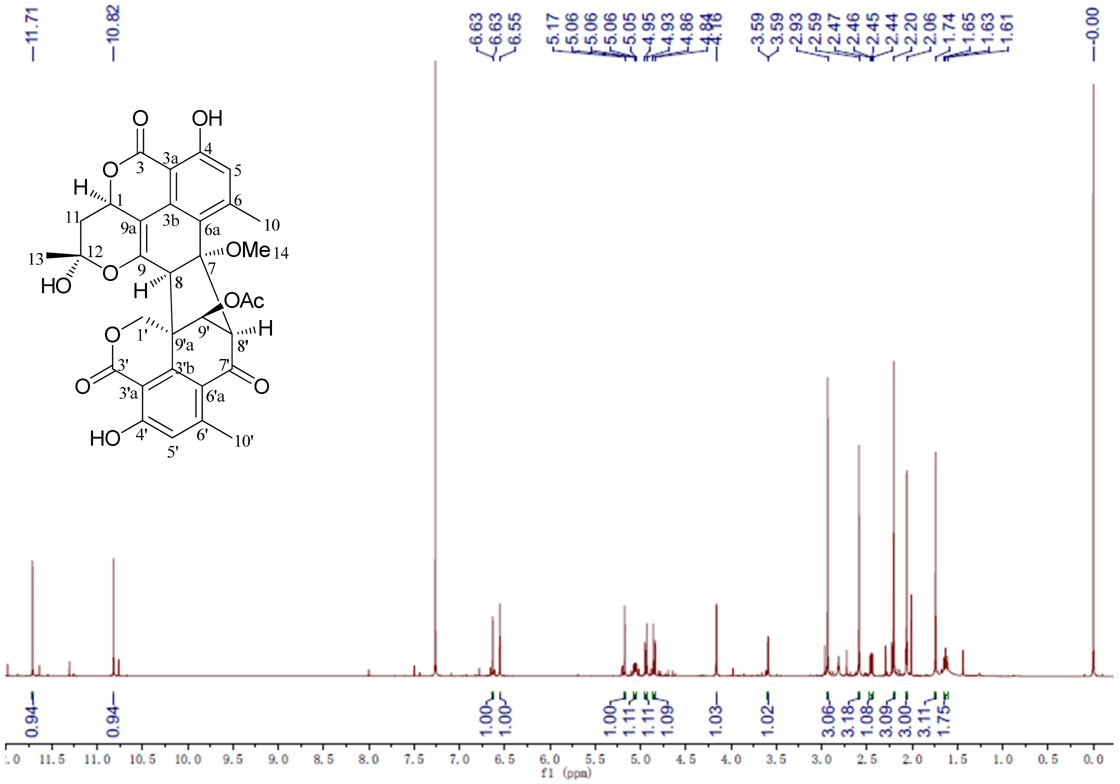

S4: ^{13}C NMR (150 MHz, $CDCl_3$) and DEPT spectra of verruculosin A (1).

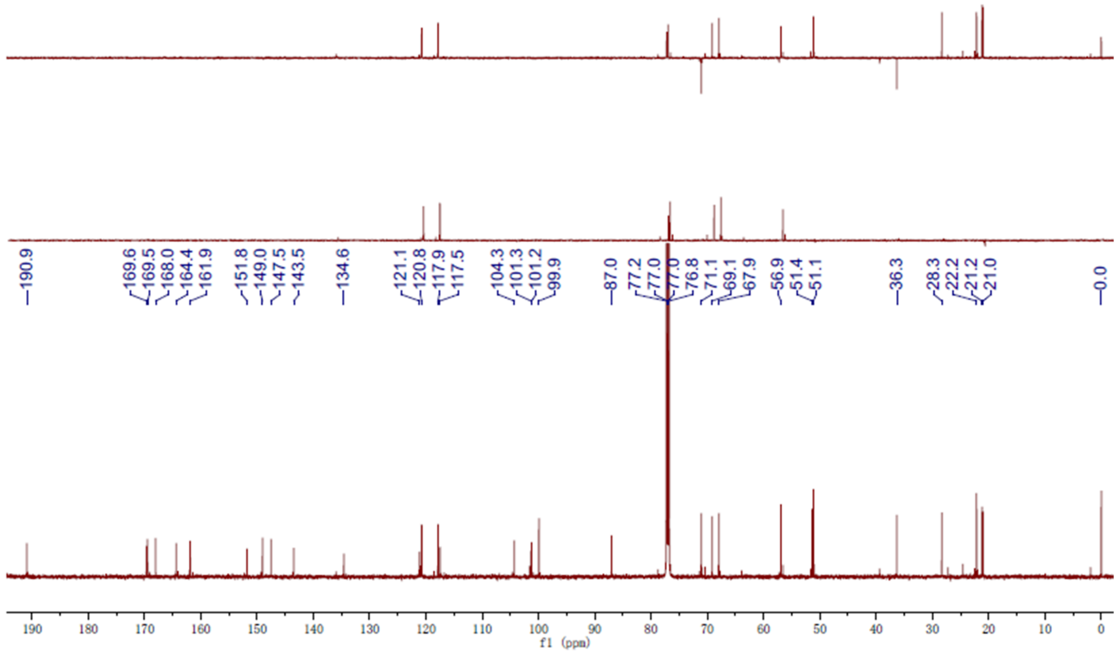

S5: Expanded ^{13}C NMR (150 MHz, $CDCl_3$) and DEPT spectra of verruculosin A (1).

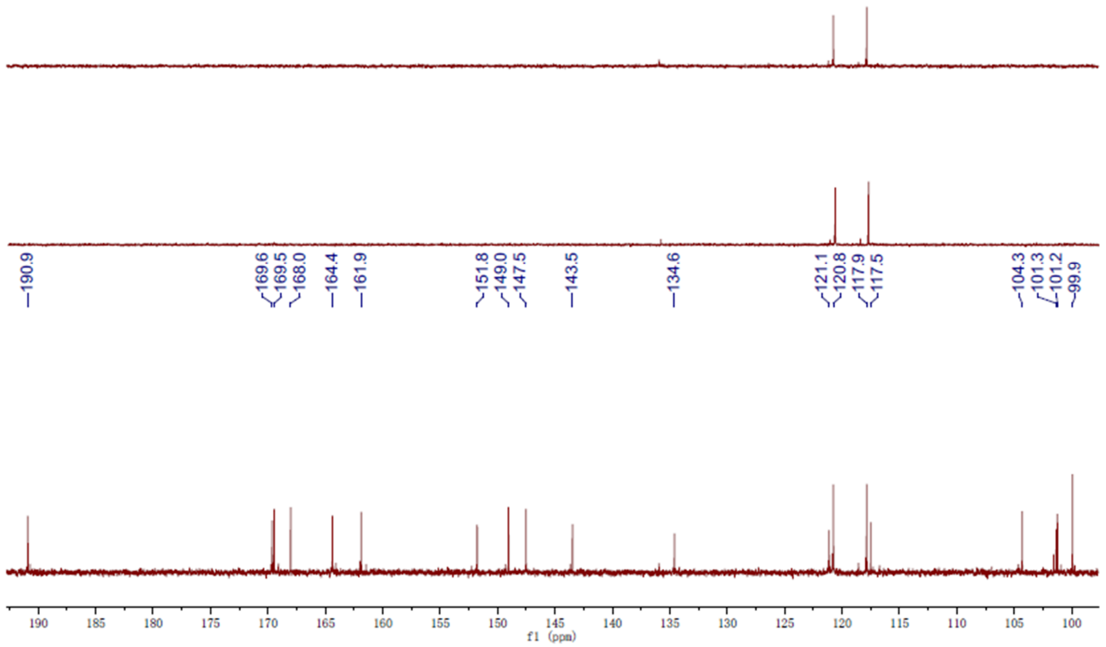

S6: Expanded ^{13}C NMR (150 MHz, $CDCl_3$) and DEPT spectra of verruculosin A (1).

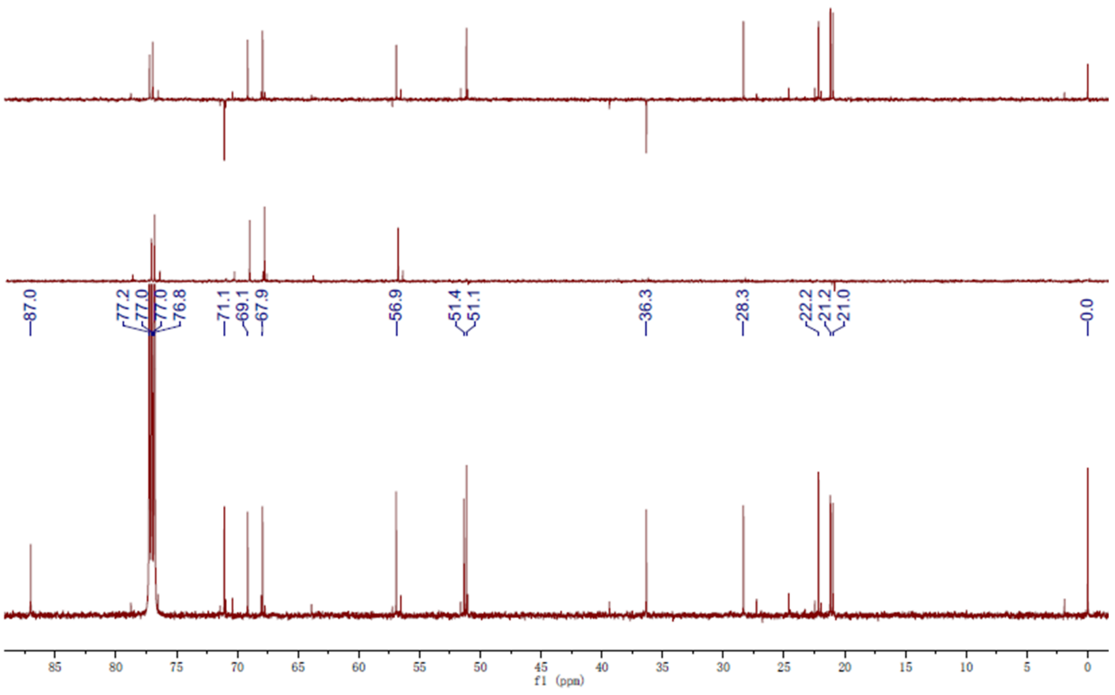

S7: ^{1}H-^{1}H COSY NMR spectrum of verruculosin A (1).

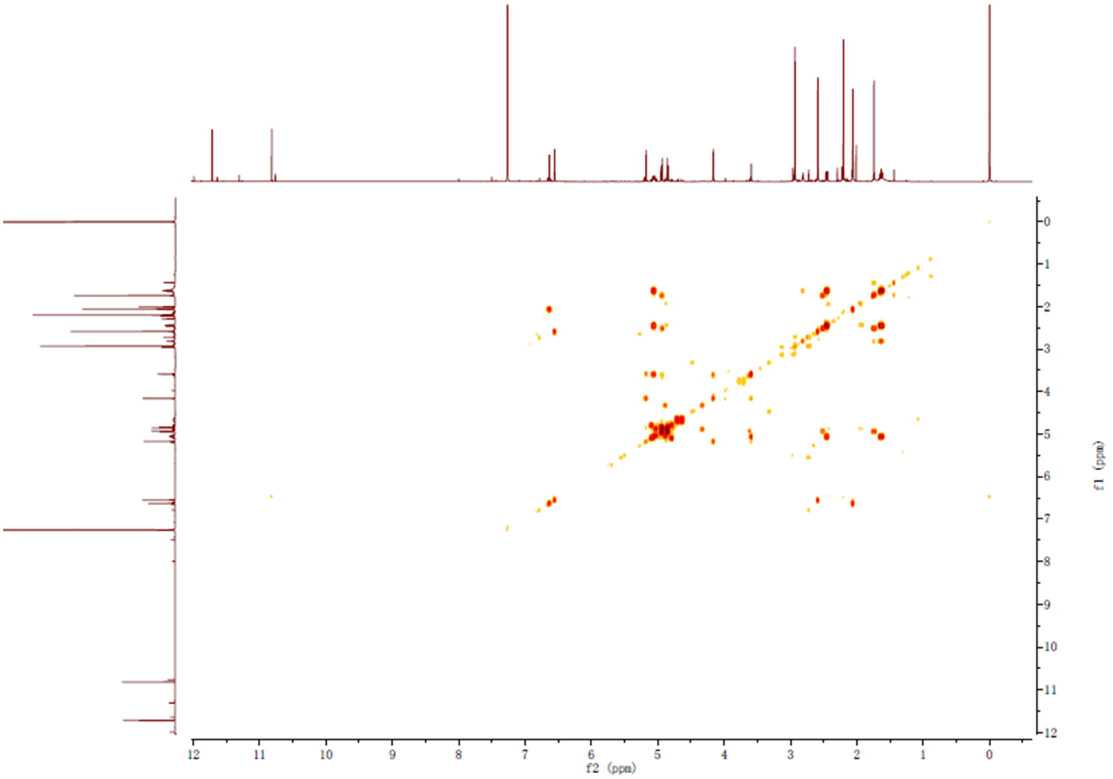

S8: HSQC NMR spectrum of verruculosin A (1).

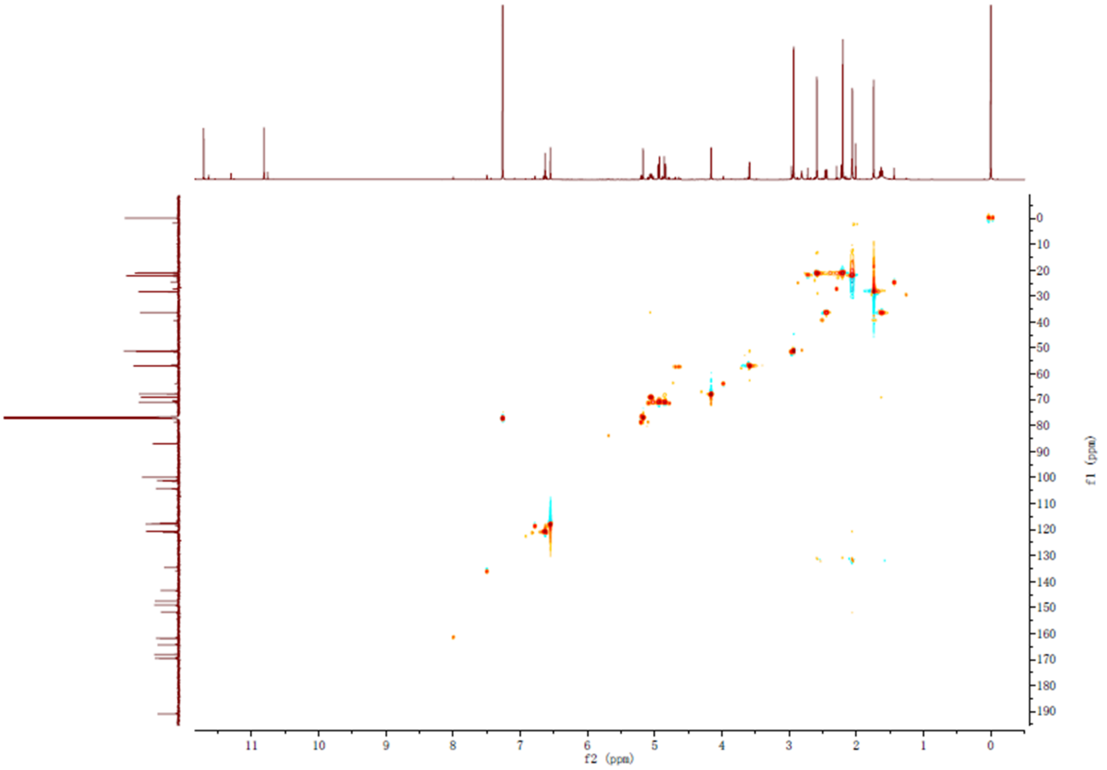

S9: HMBC NMR spectrum of verruculosin A (1).

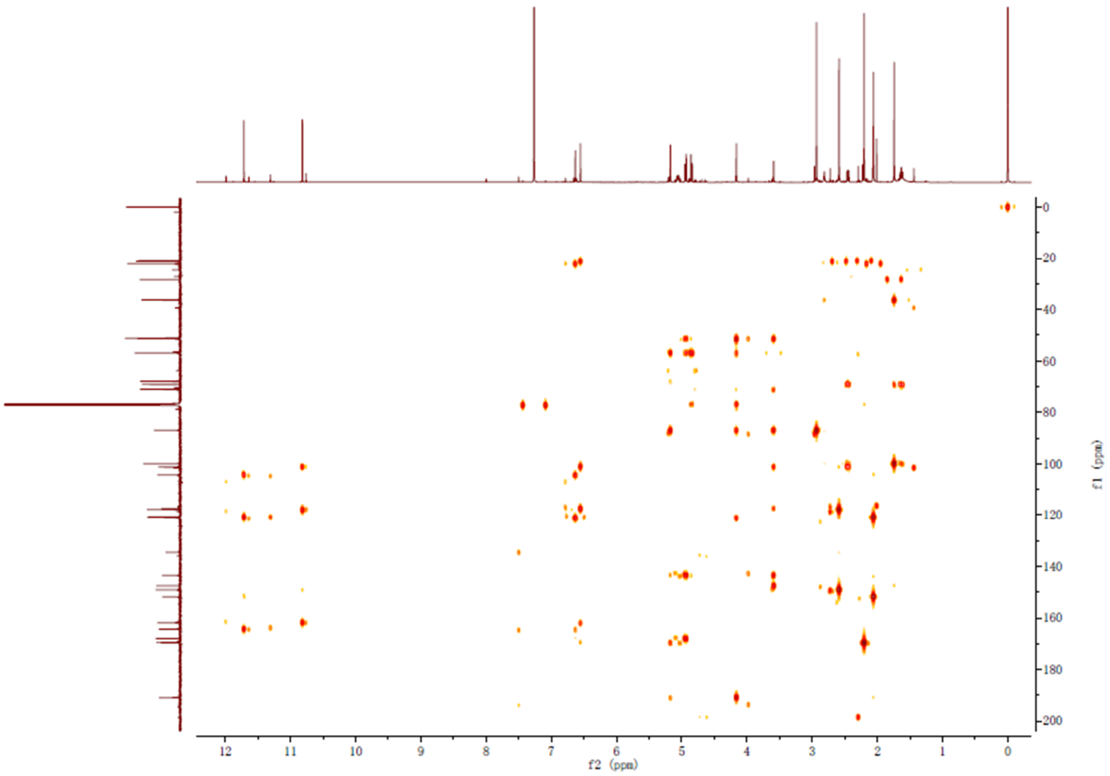

S10: NOESY NMR spectrum of verruculosin A (1).

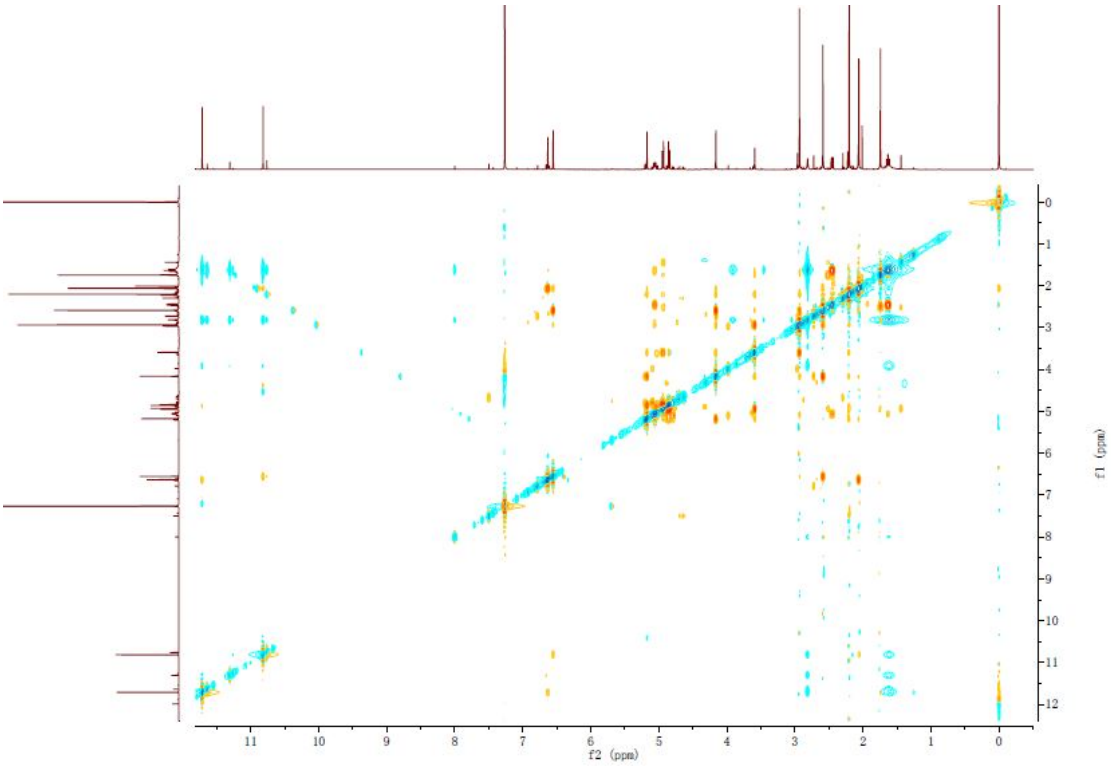

S11: ^{1}H NMR (600 MHz, CDCl3) spectrum of verruculosin B (2).

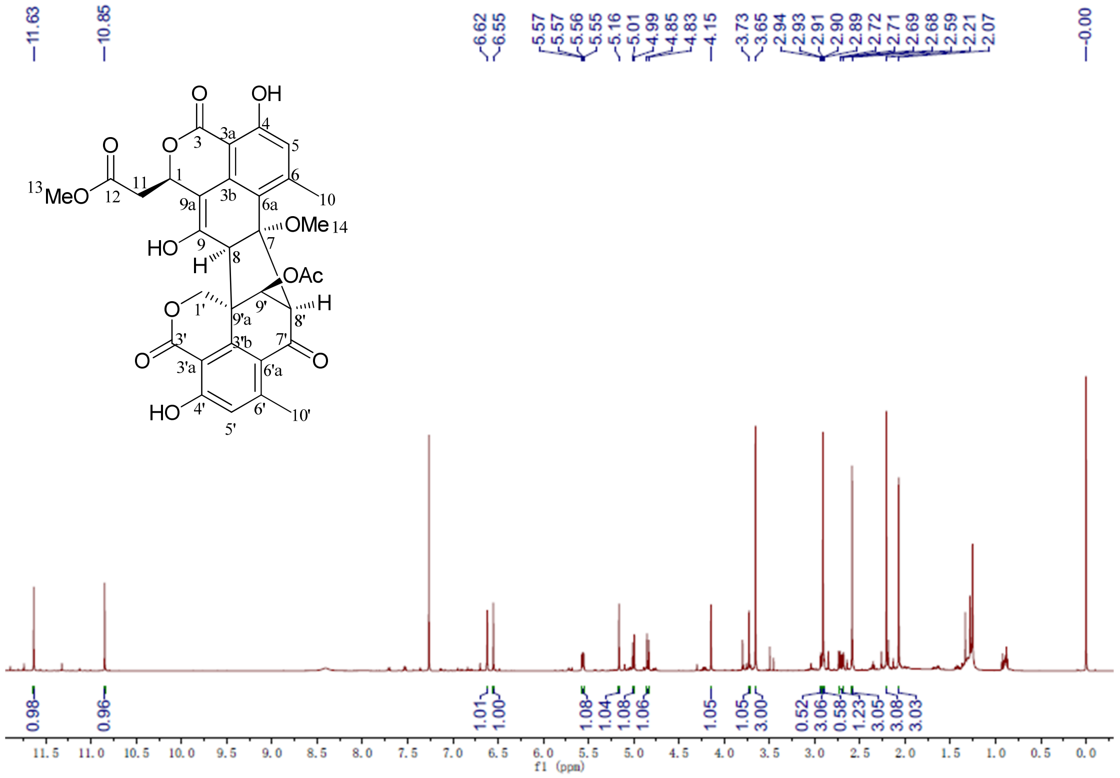

S12: ^{13}C NMR (150 MHz, CDCl3) and DEPT spectra of verruculosin B (2).

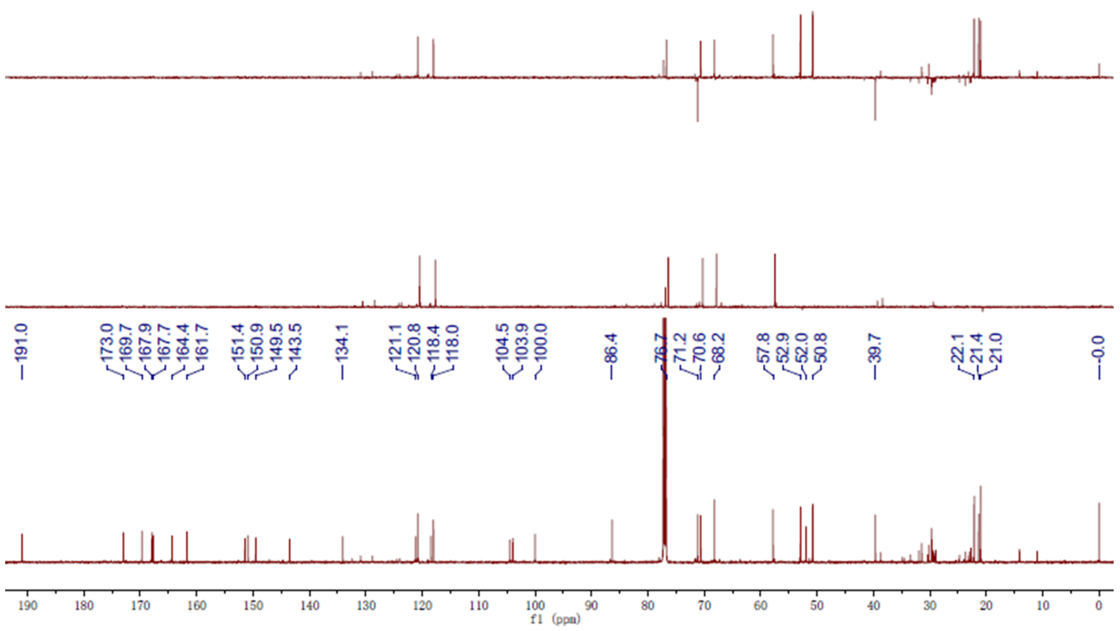

S13: ^{1}H-^{1}H COSY NMR spectrum of verruculosin B (2).

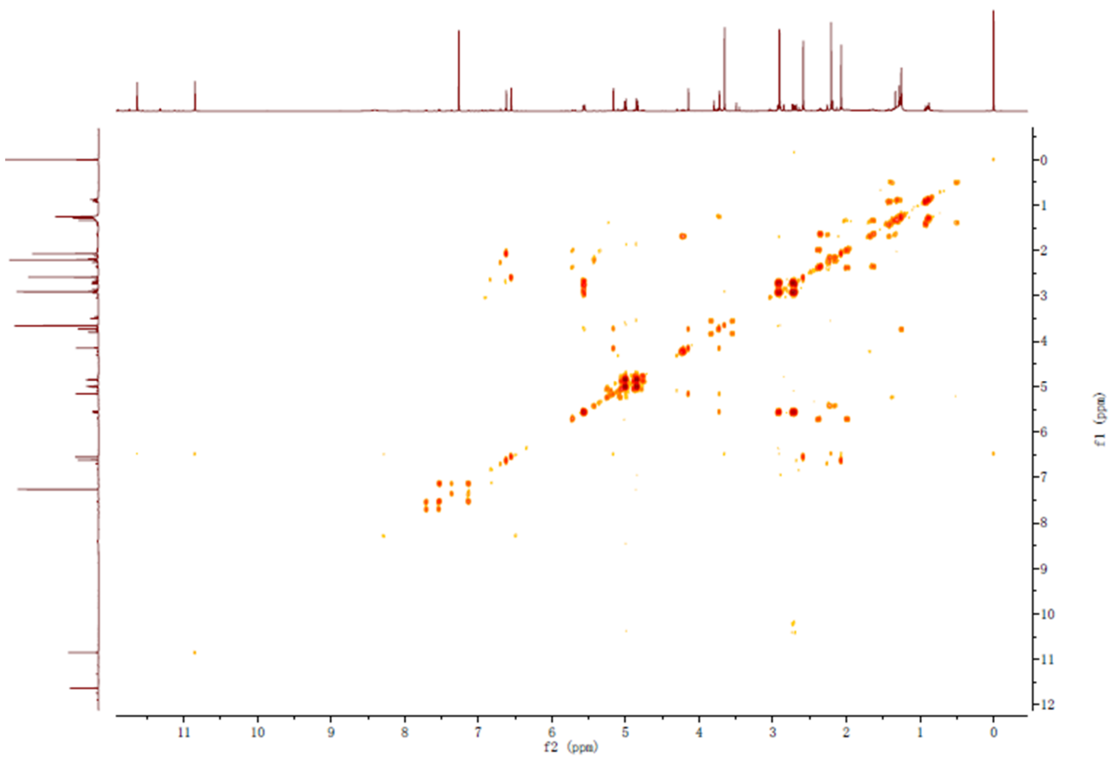

S14: HSQC NMR spectrum of verruculosin B (2).

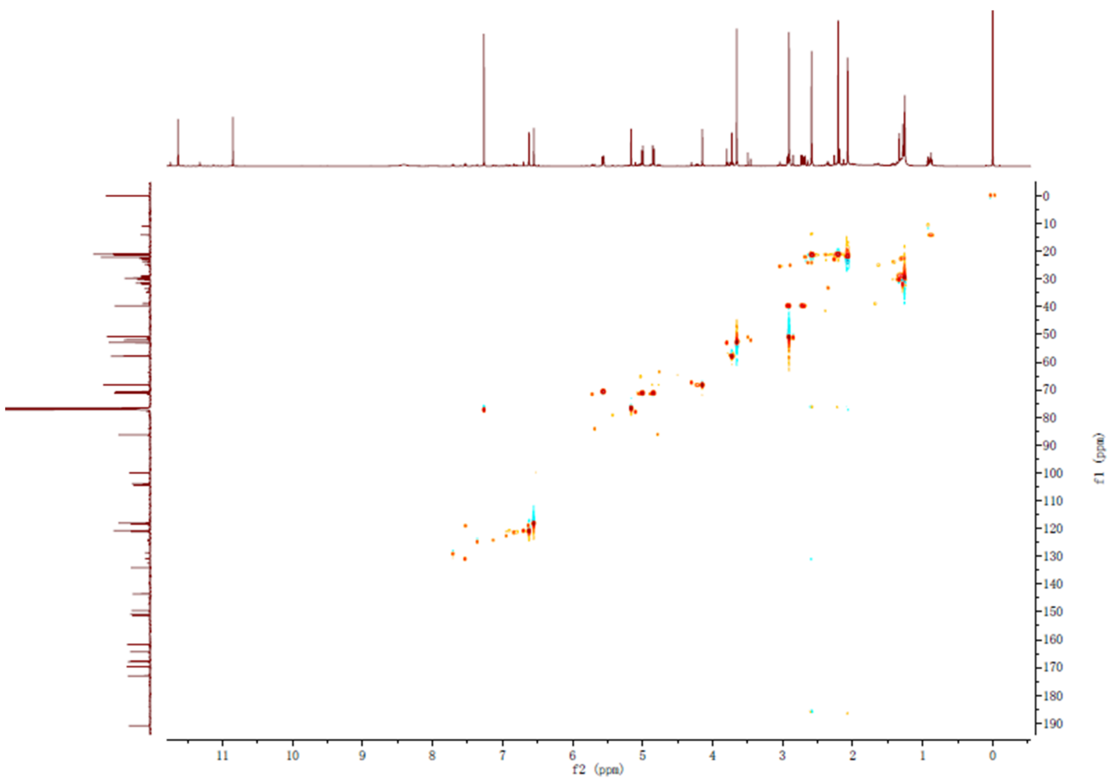

S15: HMBC NMR spectrum of verruculosin B (2).

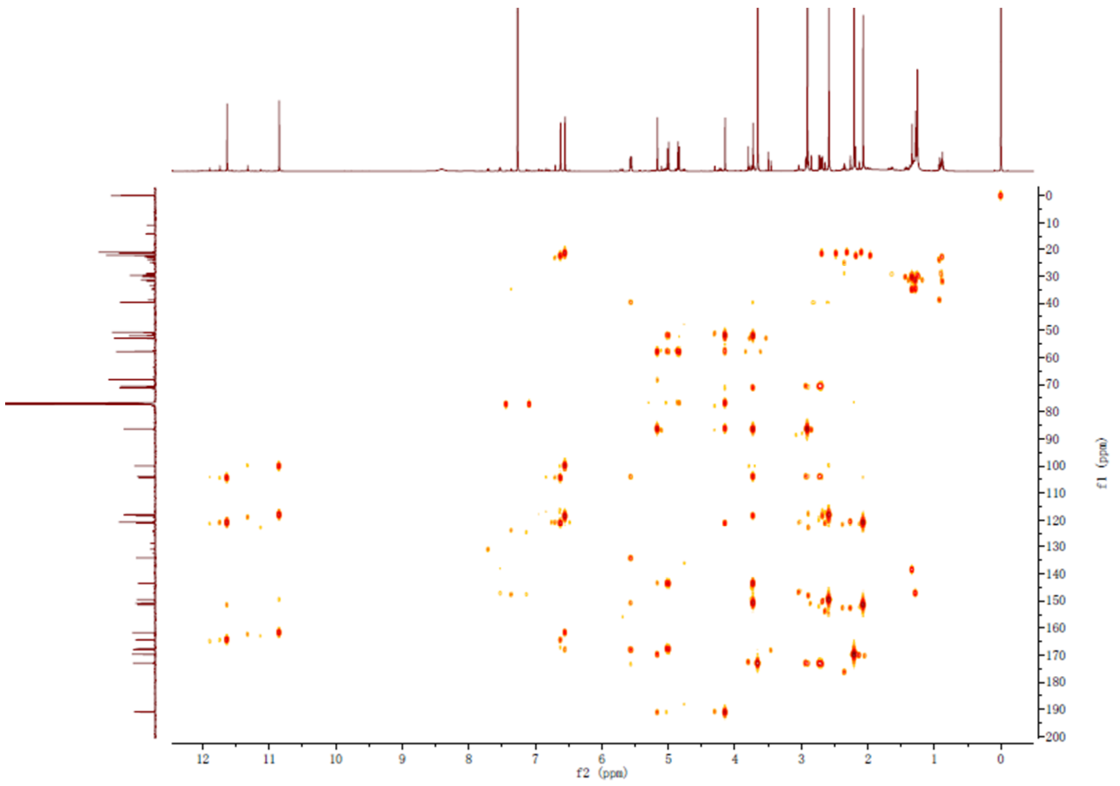

S16: NOESY NMR spectrum of verruculosin B (2).

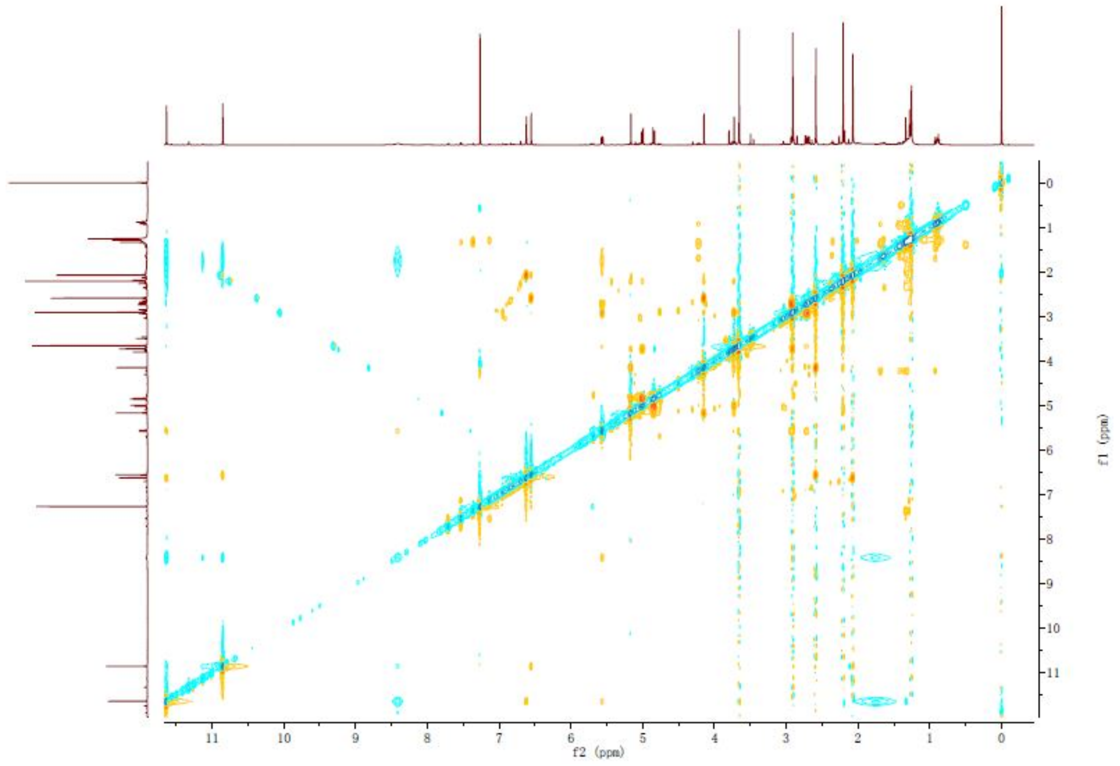

S17: ^{1}H NMR (600 MHz, $CDCl_3$) spectrum of verruculosin C (3).

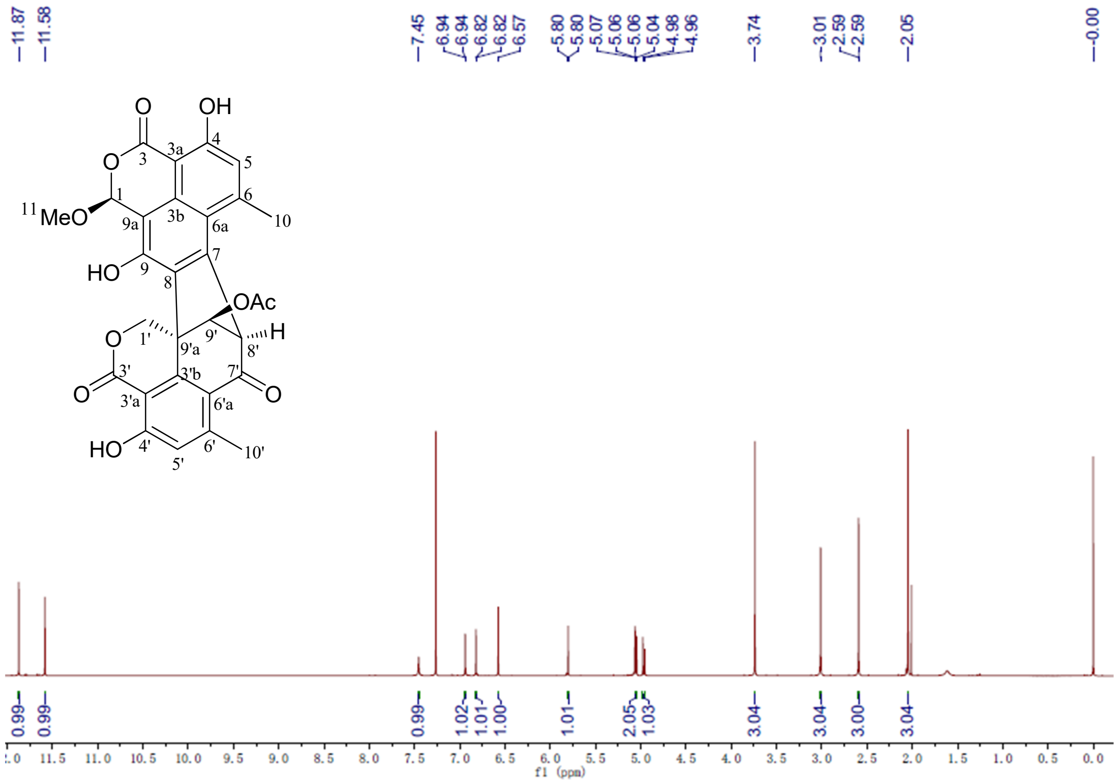

S18: ^{13}C NMR (150 MHz, $CDCl_3$) and DEPT spectra of verruculosin C (3).

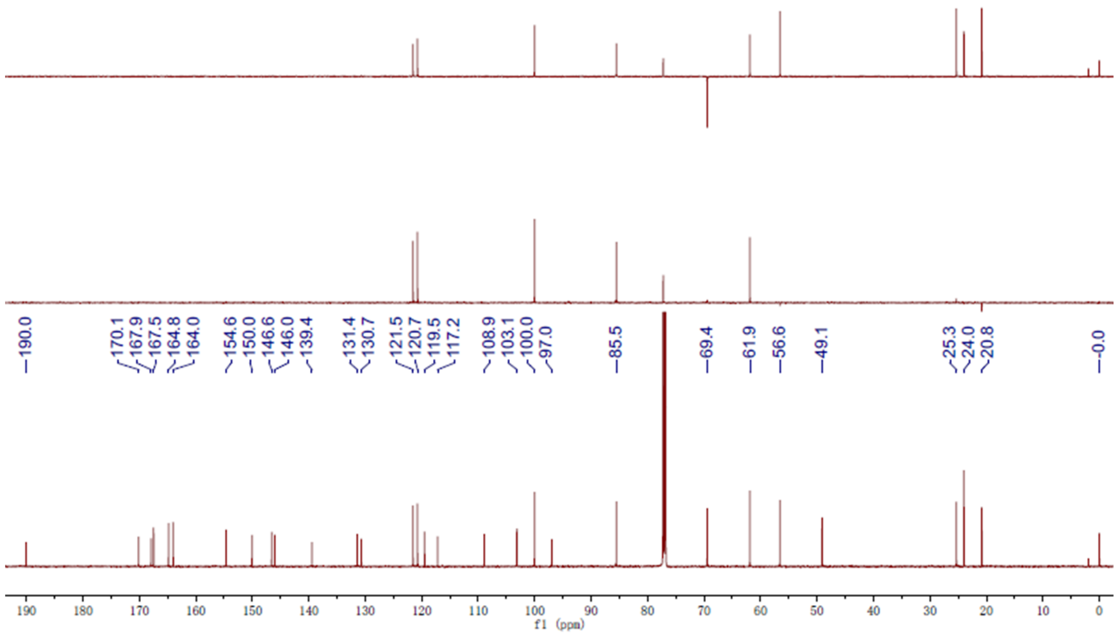

S19: ^{1}H-^{1}H COSY NMR spectrum of verruculosin C (3).

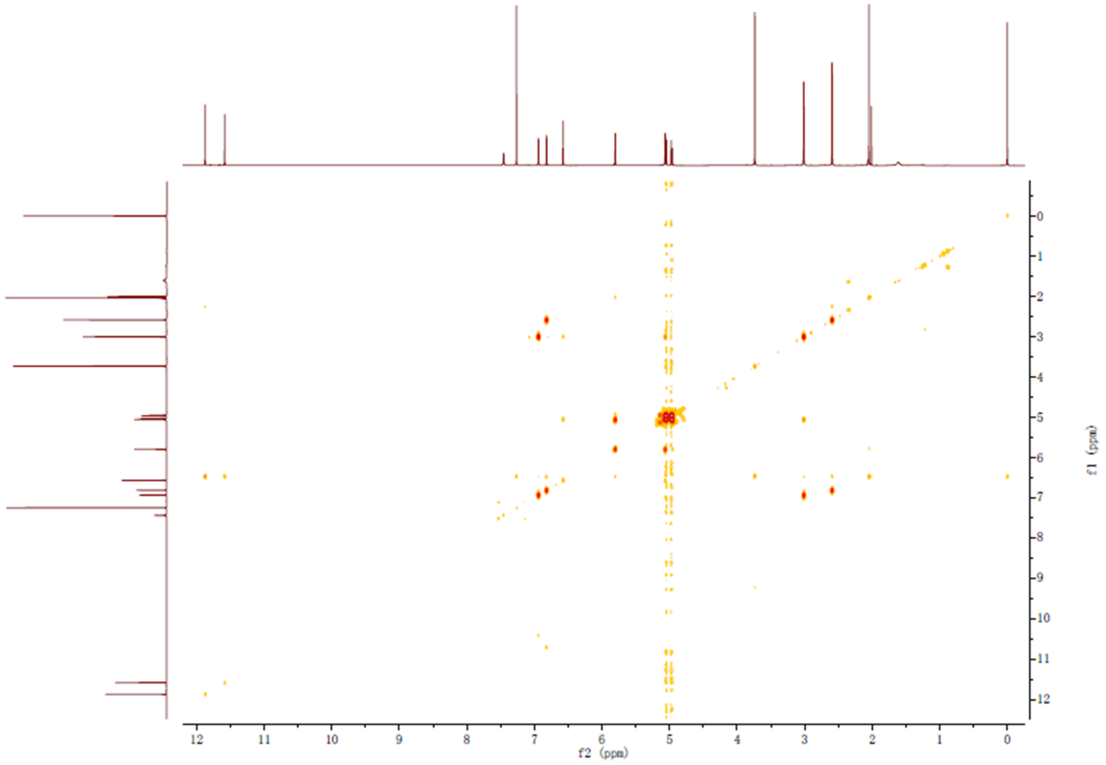

S20: HSQC NMR spectrum of verruculosin C (3).

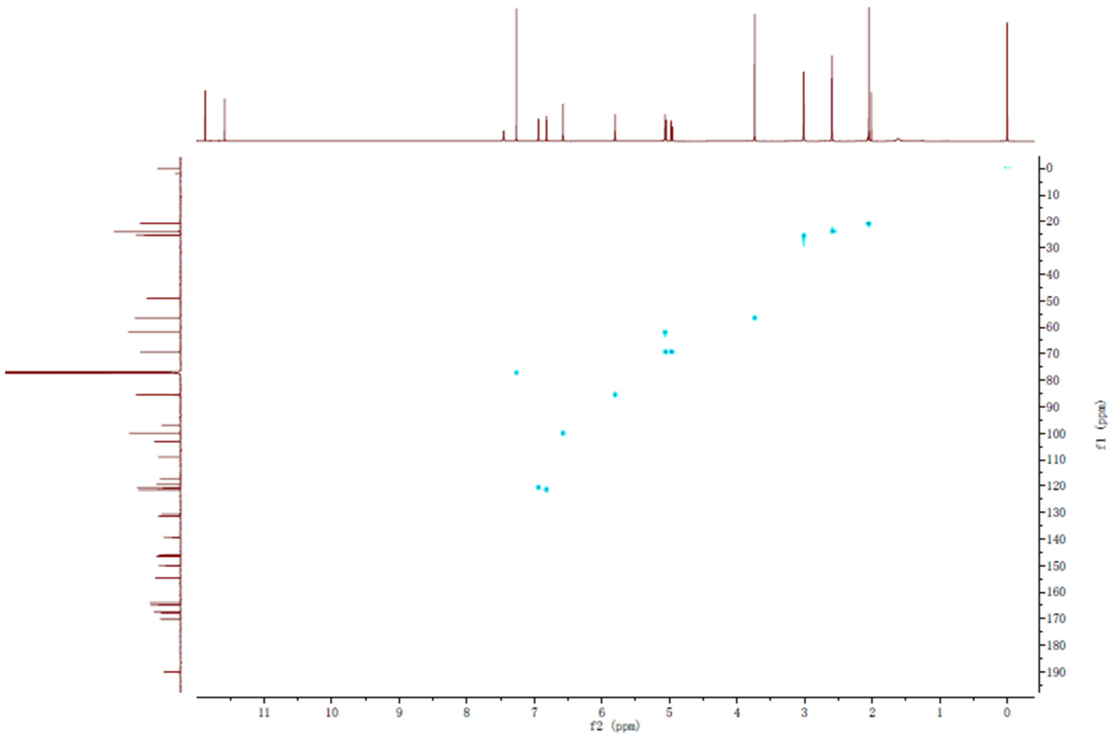

S21: HMBC NMR spectrum of verruculosin C (3).

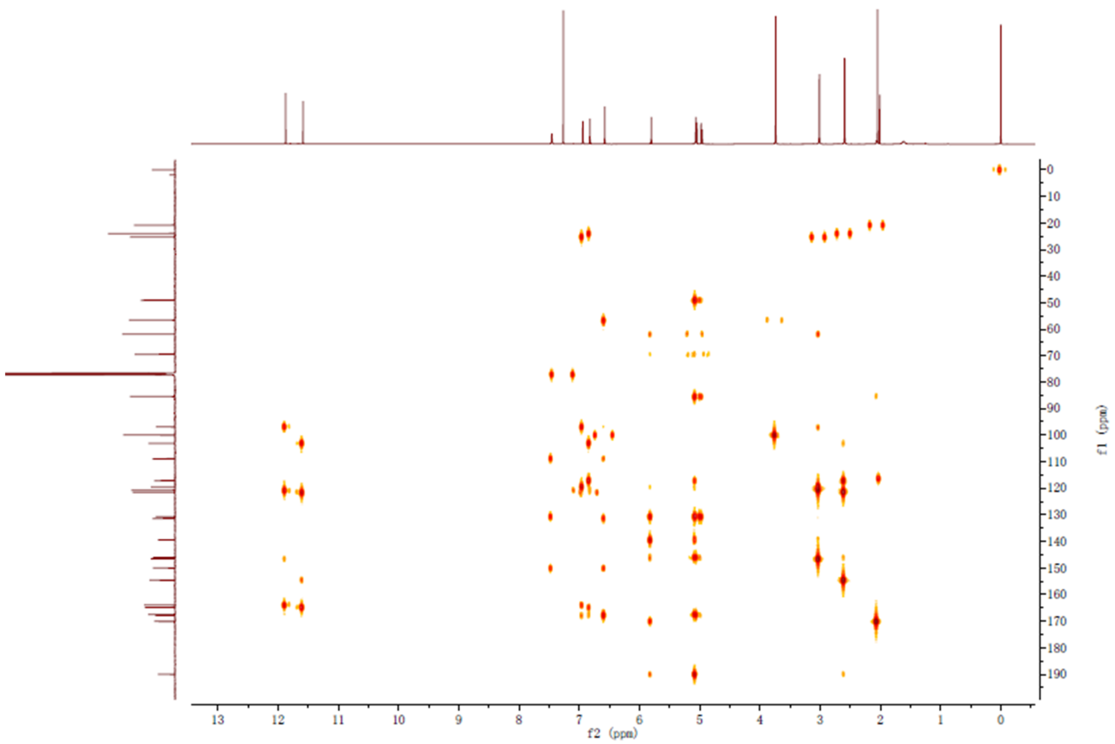

S22: NOESY NMR spectrum of verruculosin C (3).

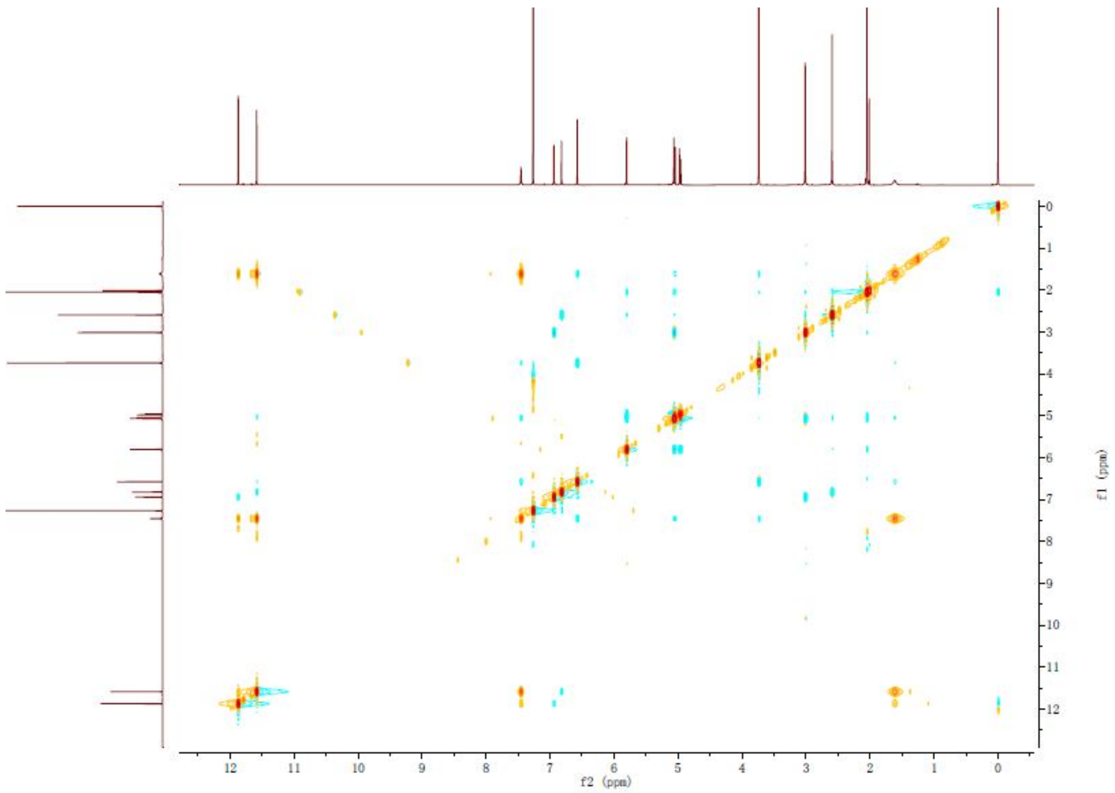

Supplement: Supplementary file 1 [file marinedrugs-17-00516-s001.zip › marinedrugs-582687-SI.pdf]
